# Engineering rhodium encapsulated indium doped fullerene for NH_3_, NO, and NO_2_ sensing

**DOI:** 10.1038/s41598-025-93796-7

**Published:** 2025-12-24

**Authors:** Adebayo P. Adeleye, Alpha O. Gulack, Lubem Aondoakaa

**Affiliations:** 1 Department of Chemistry, Marquette University, Milwaukee, WI 53233 USA; 2 Department of Chemistry, Federal University of Technology, Akure, Akure, Nigeria; 3 Department of Science Laboratory and Technology, University of Calabar, Calabar, Nigeria; 4 Department of Chemistry, University of Calabar, Calabar, Nigeria

**Keywords:** Fullerene, Adsorption, Sensor mechanism, QTAIM, DFT, Sensors, Theory and computation, Carbon nanotubes and fullerenes

## Abstract

Nitrogenous gas pollutants, such as NH_3_, NO, and NO_2_, contribute significantly to environmental degradation, driving water pollution, biodiversity loss, and impaired air quality while posing critical risks to human health. Despite advancements in gas sensing technologies, materials with enhanced sensitivity and selectivity remain crucial for reliable pollutant detection. In this work, we investigate the gas sensing properties of rhodium-encapsulated indium-doped carbon-based fullerene (In-Rh@C_60_) via DFT/PW6B95-D3/GenECP and ωB97X-D/LANL2DZ computational methods. The energy gap values were found to range from 0.705 to 1.537 eV and 3.980 to 5.166 eV for the respective methods. Upon adsorption of NH_3_, NO, and NO_2_, the energy gap decreases, indicating enhanced sensitivity. The observed chemisorption phenomena exhibit adsorption energies between -13.49 and -8.397 eV. Notably, adsorption of NO at the O-site and N-site leads to the most pronounced energy gap reductions (-0.515 eV and -0.389 eV). The green regions observed in the 3D non-covalent interaction plots signify strong van der Waals interactions, contributing to the stability of the adsorbent-adsorbate systems. This tailored system demonstrates suitability as an adsorbent material for gas pollutants, with adsorption site specificity taken into account. The findings suggest that the modified In-Rh@C_60_ system holds potential as an adsorbent material for integration into sensor devices aimed at detecting NH_3_, NO, and NO_2_ gas pollutants.

**Supplementary Information:**

The online version contains supplementary material available at 10.1038/s41598-025-93796-7.

## Introduction

Air pollution, whether from natural or anthropogenic sources, remains a major threat to both human health and the environment. Toxic gases, particularly nitrogen-containing gases such as ammonia (NH_3_), nitric oxide (NO), and nitrogen dioxide (NO_2_), have been linked to respiratory irritation, eye and skin irritation, and chronic respiratory diseases, including asthma and bronchitis^1^. NH_3_, a colorless gas with a pungent odor, is predominantly emitted through agricultural activities such as fertilizer application and livestock waste management^2^. The release of NH_3_ from these agricultural sources not only pollutes water and soil but also contributes to eutrophication and soil acidification, ultimately exacerbating respiratory health risks for humans^3–5^. NO, a reactive gas primarily released from high-temperature combustion processes, including power plants and motor vehicle engines, is relatively short-lived in the atmosphere but plays a critical role in air pollution. It readily oxidizes to form NO_2_ and contributes to the formation of ground-level ozone and photochemical smog, both of which are associated with adverse respiratory effects^6,7^. NO_2_, which is also directly emitted from combustion sources such as vehicular exhaust and industrial emissions, further worsens air quality by contributing to acid rain and widespread environmental degradation. In addition to its environmental impacts, exposure to NO_2_ has been strongly linked to respiratory ailments such as asthma, bronchitis, wheezing, and coughing^6–10^. Prolonged exposure to elevated levels of these toxic gases results in severe health consequences, including respiratory and cardiovascular diseases, neurological disorders, and even mortality^11–13^. Given their prevalence and detrimental effects, the development of high-performance gas sensors is critical. Such sensors are indispensable for detecting toxic gases at trace levels, enabling early intervention and the prevention of environmental and industrial hazards.

Remarkably, nanomaterials have gained significant attention for their potential in high-performance gas sensors. Some of which include monolayers, nanosheets, nanotubes, and nanowires^14–20^. Among the widely explored nanomaterials, fullerenes could be considered highly promising due to their unique properties and relevance to environmental remediation. Fullerenes represent a fascinating class of carbon molecules characterized by hollow structures, including spheres, ellipsoids, or tubes, composed entirely of carbon atoms. Among these, C_60_, the most studied and well-recognized fullerene, consists of 60 carbon atoms arranged in a structure resembling a soccer ball^21^. The discovery of fullerenes in 1985 by Harold Kroto, Robert Curl, and Richard Smalley marked a significant milestone in the field of nanotechnology^21,22^. Since then, fullerenes have sparked immense interest across diverse disciplines, including materials science, chemistry, physics, and biomedicine^23^. What sets fullerenes apart from other carbon allotropes are their extraordinary physical and chemical properties. Their high mechanical strength, excellent electrical conductivity, remarkable thermal stability, and notable electron affinity position them as indispensable materials in sensor technology. These attributes also underpin their applications in a variety of fields, such as nanoscale sensors, organic photovoltaics, energy storage, electronics, drug delivery, and catalysis^24–27^.

In recent years, their use in gas sensing has gained momentum due to their high surface area, chemical stability, and tunable electronic properties^28^. Studies have demonstrated the effectiveness of fullerene-based materials in detecting toxic gases, notably volatile organic compounds (VOCs), hydrogen sulfide (H_2_S), nitrogen oxides (NOx), and sulfur dioxide (SO_2_)^29^. Gakhar et al.^30^ reported the development of a C_60_-TiO_2_ sensor for the detection of VOCs in trace amounts. The sensor demonstrated outstanding sensitivity to formaldehyde compared to other VOCs. Decorating TiO_2_ nanotubes with functionalized C_60_ markedly enhanced their Brunauer–Emmett–Teller (BET) surface area relative to pure TiO_2_ nanotubes. Furthermore, the combination of fullerenes with other nanomaterials, such as metal oxides, quantum dots, and polymers, has been explored to enhance the sensing properties and operational stability of gas sensors^31^. Recent advancements in computational techniques and quantum mechanical simulations have also facilitated the understanding of the interactions between gas molecules and fullerene-based nanostructures, providing valuable insights into the underlying mechanisms governing the gas sensing behavior^32–34^. Keshekar et al.^35^, combining experimental and computational techniques, investigated the gas sensing properties of a novel nanohybrid of SnO_2_ quantum dots-fullerene (SnO_2_ QDs-C_60_) for the detection of H_2_S, CH_4_, and C_3_H_8_. At 100–200 °C, nanohybrid showed excellent selectivity and response for H_2_S at 70 ppm compared to 1% CH_4_ and 1% C_3_H_8_. Density functional theory (DFT) calculations supported these findings, identifying H_2_S as having the strongest interaction with the SnO_2_-C_60_ hybrid through significant band-gap variation. Hung-Bin Lin and Jeng-Shong Shih^36^ developed a C_60_-cryptand 22 (C_60_-di-propylamine-cryptand 22) coated surface acoustic wave (SAW) quartz crystal sensor. This design showed enhanced sensitivity to both polar and nonpolar organic molecules, surpassing the individual performance of C_60_ or cryptand 22. With detection limits of 0.1–3.0 mg/ml, the SAW sensor also demonstrated comparable efficacy to commercial thermal conductivity detectors (TCDs) for various organic compounds. Similarly, Smazna et al.^37^ designed a C_60_-decorated ZnO tetrapod material, revealing significant charge transfer and bandgap modulation between C_60_ and ZnO through electron microscopy and spectroscopy. This hybrid displayed promising ethanol gas sensing capabilities, with enhanced ZnO bandgap adsorption and visible absorption bands proportional to C_60_ concentration.

Recent DFT investigations by Abbasi et al.^38^ have demonstrated the gas-sensing potential of Pd-decorated stanene monolayers through their interactions with CO, NO, N₂O, and NH₃ molecules. Among three evaluated Pd embedding patterns, hollow-site decoration was identified as the most energetically favorable, with an adsorption energy of -6.80 eV. Charge density analysis revealed significant electron accumulation on the gas molecules and strong covalent bonding with the embedded Pd atoms, particularly for CO, which exhibited enhanced semiconductor properties and the largest band gap among the studied gases. These findings highlight the strong chemisorption interactions and selective gas sensing capabilities of Pd-decorated stanene monolayers.

Fullerene-based materials have also been explored for their drug delivery applications. Recently, Moztarzadeh and co-workers^39^ investigated the physicochemical properties of a novel conjugated fullerene with nitric oxide [C_60_ + NO] system using density functional theory and time-dependent density functional theory. They showed that the system exhibited enhanced electrical and optical characteristics, with a bipolar moment of approximately 12.92 D and reduced HOMO or LUMO energy levels compared to free C_60_. Further studies are warranted in tissue cultures as the system demonstrates the potential for various biomedical applications, such as targeted drug delivery, therapy, and imaging. A unique combination of sensitivity and selectivity of fullerene-based sensors make them valuable for applications in public health, industrial safety, and environmental monitoring. Research into the development of high-performance gas sensors based on fullerenes and their derivatives is still ongoing due to the growing significance of gas detection and monitoring^40^. In this study, we employed DFT calculations to investigate the electronic properties and interactions of In-Rh@C_60_ with toxic gases, including NH_3_, NO, and NO_2_. The novelty of this research lies in the unique design of a fullerene system doped with indium (In) and encapsulating rhodium (Rh), which, to the best of our knowledge, has not been previously explored for the adsorption of the selected gas molecules. Our primary objective is to evaluate the effectiveness of the engineered system as a sensitive and selective sensor for detecting these targeted gases.

## Computational methodology

The adsorption of NH_3_, NO_2,_ and NO gas molecules on the In-Rh@C_60_ system was examined using the DFT methods at the DFT/PW6B95-D3/GenECP and ωB97X-D/LANL2DZ levels of theory. The Gaussian 16 program^41^ was used for the computational modeling. Due to the presence of heavy metals (In and Rh), the LanL2DZ basis set was used in the calculations, as they are known for accurately predicting adsorption processes^43^. Before optimization, all structures were built and visualized using the Gauss View 6.0.16 package^42^ and CYLview visualization and analysis software for stable conformation^44^. Their geometries and electronic properties were obtained, visual studies were conducted, and the sensing mechanisms were identified via optimization. The adsorption energy for all studied systems was calculated using the Eq. (1) as follows:

1 $$\:{E}_{ads}={E}_{system}-\left({E}_{surface}+{E}_{molecule}\right)$$

Where, $\:{E}_{molecule}$, $\:{E}_{surface}$, and $\:{E}_{system}\:$represent the energies of the adsorbate (NH_3_, NO, and NO_2_), the modified system (In-Rh@C_60_), and the gas-adsorbed system, respectively. The visual analyses, including density of states (DOS), electron localization function (ELF), non-covalent interaction (NCI), and quantum theory of atoms in molecules (QTAIM), as well as the d-band center calculation, were performed using the Multiwfn 3.7 program and plotted using the Visual Molecular Studio (VMD) software package^45,46^. The DOS plots were visualized using Origin software. Finally, the Chemcraft 1.6 program^47^ generated both the HOMO and LUMO plots.

## Results and discussion

### Structural geometry

**Fig. 1:**
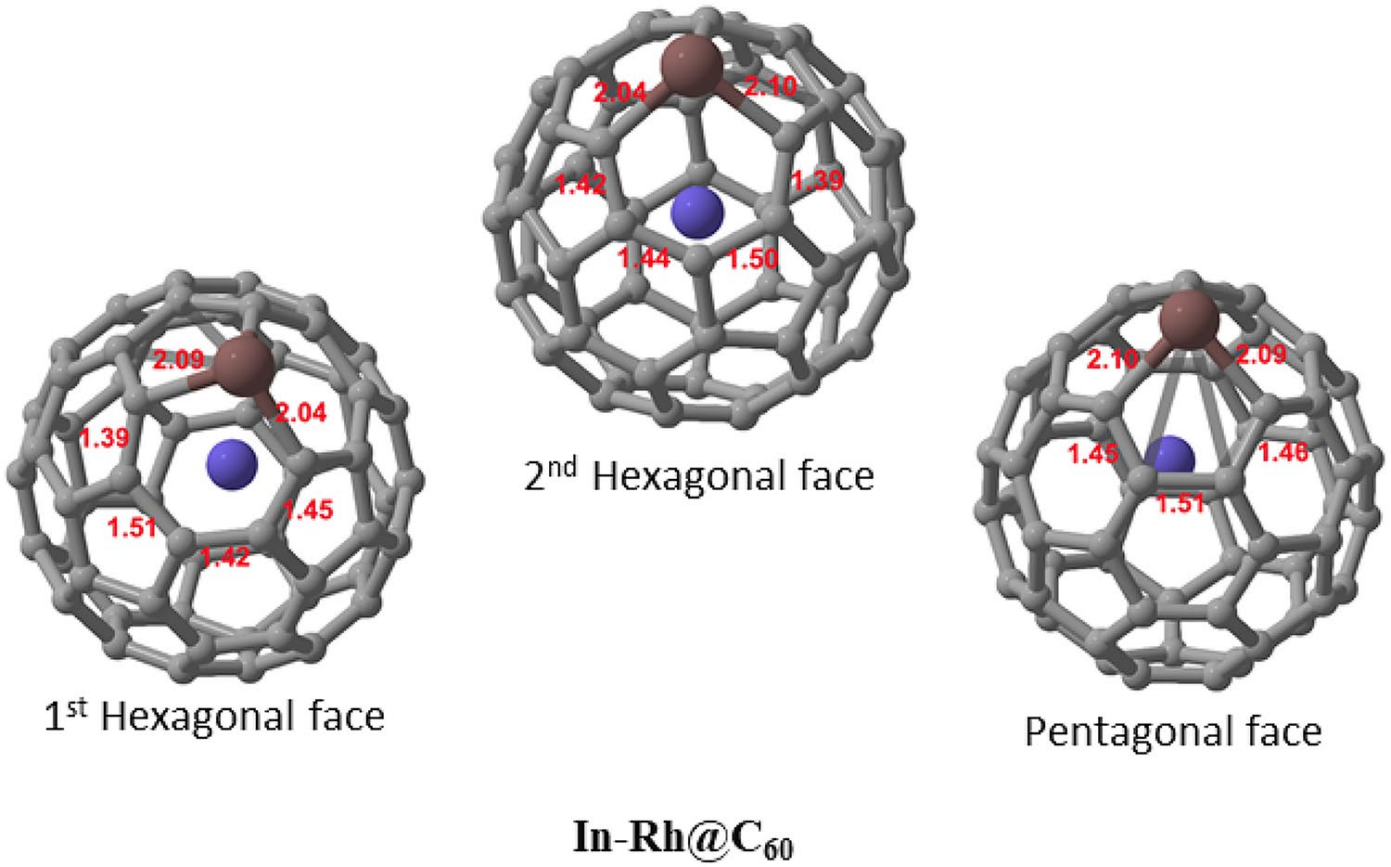
Optimized structures of the modified In-Rh@C_60_ system showing the first and second hexagonal faces, as well as the pentagonal face.

The optimized molecular structure of In-Rh@C_60_ is depicted in Fig. 1. Fullerene C_60_ is a three-dimensional (3D) carbon structure that consists of 60 covalently bonded carbon atoms arranged in a spherical shape. It is made up of 12 pentagonal faces and 20 hexagonal faces which allow C_60_ to form a stable, cage-like structure. Herein, the C_60_ nanocage was structurally doped with In, while Rh was encapsulated within its hollow cage structure. These structural modifications influence the system’s electronic properties and its nature of interactions with gas molecules, such as NH_3_, NO, and NO_2_ upon adsorption. The C-In bond lengths in the optimized structure are 2.09 Å, 2.04 Å, and 2.10 Å, with an average of 2.08 Å. The pentagon face of the system exhibits C-C bond lengths of 1.45 Å, 1.51 Å, and 1.46 Å, resulting in an average of 1.47 Å. Meanwhile, the average C-C bond lengths in both the first and second hexagonal faces adjacent to the In atom are 1.44 Å.

**Fig. 2:**
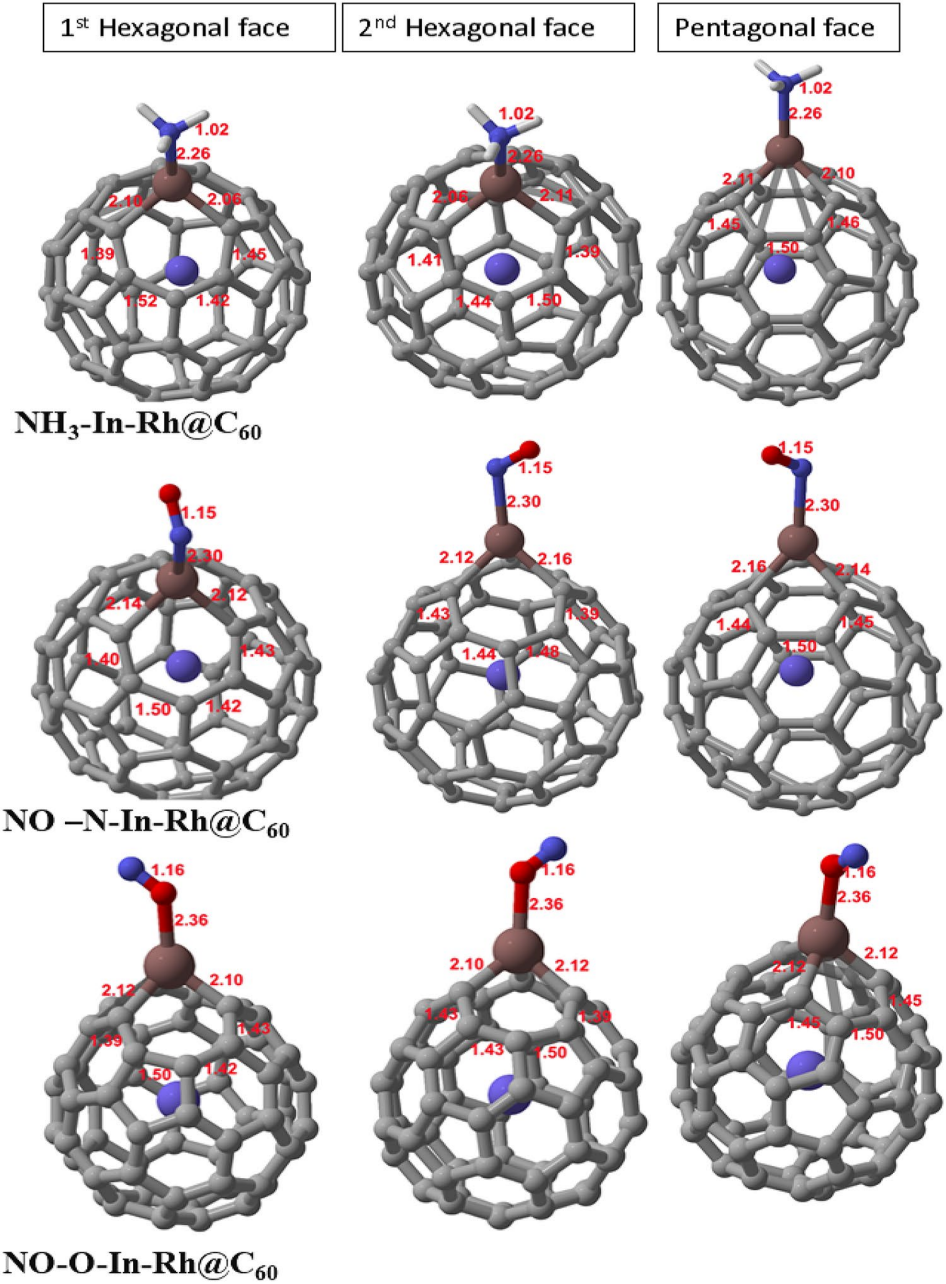
Optimized structures of the gas-adsorbed In-Rh@C_60_ systems upon interaction with NH_3_ and NO (with two adsorption sites considered), showing the first and second hexagonal faces, as well as the pentagonal face.

**Table 1:** Average C-In and C-C bond lengths bond lengths of the first and second hexagonal faces and the pentagon face adjacent to the N- and O- adsorption sites of the modified In-Rh@C_60_ system and the corresponding gas-adsorbed systems.

System	Adsorption site C-In bond length (Å)	1st hexagonal face C-C bond length (Å)	2nd hexagonal face C-C bond length (Å)	Pentagon face C-C bond length (Å)
In-Rh@C_60_	2.08	1.44	1.44	1.47
NH_3__In-Rh@C60	2.09	1.45	1.44	1.47
NO-N-In-Rh@C_60_	2.14	1.44	1.44	1.46
NO-O-In-Rh@C_60_	2.11	1.44	1.44	1.47
NO_2_-N-In-Rh@C_60_	2.19	1.44	1.44	1.46
NO_2_-O-In-Rh@C_60_	2.17	1.44	1.44	1.46

As shown in **Table** 1, upon adsorption of NH_3_, the average C-In bond length in the NH_3_-In-Rh@C_60_ system measured 2.09Å, indicating no significant deviation from the modified system (In-Rh@C_60_). Similarly, the average C-C bond lengths of the first and second hexagonal faces were measured at 1.45 Å and 1.44 Å, respectively, while that of the pentagonal face was placed at 1.47 Å, respectively (see Fig. 2). This suggests that the interaction of NH_3_ with the system does not affect the electronic structural properties of the modified system. After the adsorption of NO at the N-site, the average C-In bond length in the NO-N-In-Rh@C_60_ system measured 2.14 Å, with an increase of 0.06 Å over that of the modified system. This indicates that a strong interaction exists between NO and In at the N-site of adsorption on the system. This can be attributed to the polarity of NO. The average C-C bond lengths of both the first and second hexagonal faces adjacent to the In atom gave a negligible difference from the original system. Similarly, that of the pentagonal face resulted in a 0.01 Å reduction from the modified system. This suggests that despite the strong interaction between NO and In, it had no significant effect on the overall carbon network of the system.

The adsorption of NO at the O-site resulted in a 0.03 Å increase in the C-In bond length, indicating an influence on the NO-O-In-Rh@C_60_ system. In atom has a low electronegativity while the nitrogen atom in NO gas is electronegative. The resulting polarization of the C-In bond could rationalize the changes in the bond length. Whereas, both the hexagonal and pentagonal faces resulted in no difference, indicating that the polarization does not affect the ordered carbon network.

**Fig. 3:**
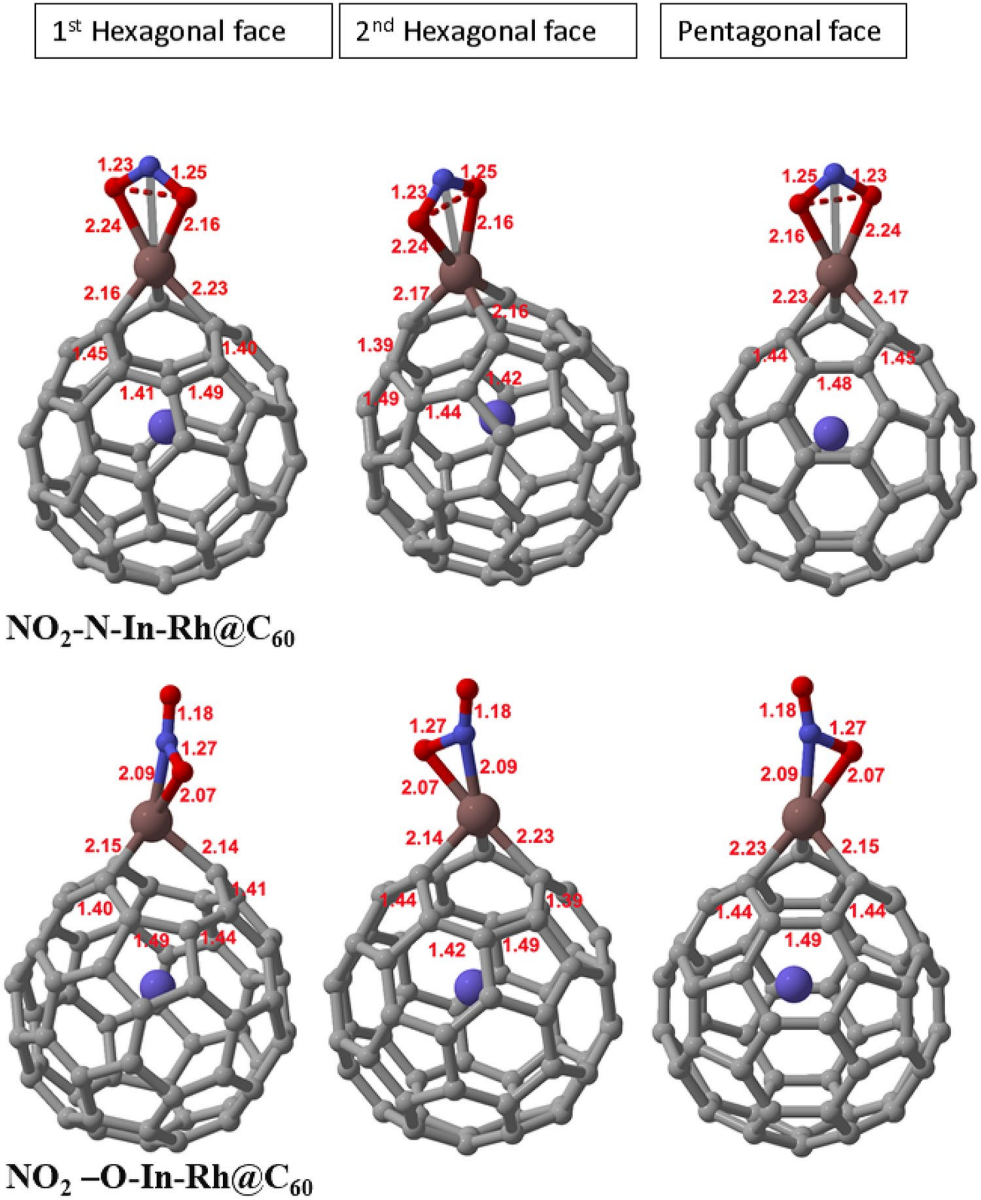
Optimized structures of the gas-adsorbed In-Rh@C_60_ systems upon interaction with NO_2_ at N and O sites, showing the first and second hexagonal faces, as well as the pentagonal face.

Furthermore, when NO_2_ was adsorbed at the N-site, the average C-In bond length resulted in a remarkable 0.11 Å increase from the original system, indicating a significant influence on the NO_2_-N-In-Rh@C_60_ system (see Fig. 3). NO_2_ has a bent molecular geometry due to the presence of a lone pair of electrons on the nitrogen atom which repels the oxygen atoms, compared to the linear molecular geometry of NO. The molecular structure and properties of NO_2_ may be responsible for the stronger interactions with In and a more significant change in the C-In bond length, compared to NO. Moreover, since it has a higher electronegativity, the resulting polarization between the nitrogen atom of NO_2_ and In may be responsible for the stronger electrostatic interactions with the site of adsorption on the system. Again, the average C-C bond lengths of both hexagonal and pentagonal faces resulted in no changes. This suggests that despite the strong interaction observed between NO_2_ and In, the carbon network of the system remained unaffected.

Additionally, upon the interaction of NO_2_ at the O-site, a 0.09 Å increase in the C-In bond length was observed, indicating a significant impact on the NO_2_-O-In-Rh@C_60_ system. Similarly, the high polarity between the electronegative nitrogen atom in NO_2_ and the relatively electropositive atom can be attributed to the strong interaction observed in the C-In bond length of the system. Meanwhile, the average C-C bond length of both the hexagonal and pentagon faces showed no significant changes in the carbon network despite the strong electrostatic interaction observed upon the adsorption of NO_2_ onto the system. Therefore, the increasing order of changes observed in the C-In bonds is as follows: NH_3_-In-Rh@C_60_ < NO-N-In-Rh@C_60_ < NO-O-In-Rh@C_60_ < NO_2_-N-In-Rh@C_60_ < NO_2_-O-In-Rh@C_60_. Overall, we observe that chemical properties, such as electronegativity and polarity, molecular structure, and atomic size play a vital role in the geometry of the In-Rh@C_60_ system upon interactions with these nitrogen-containing gases.

### Coordination and spin States stability

Evaluating the spin state and coordination environment is essential for determining the electronic structure, stability, and reactivity of metal-doped systems^48^. For the studied system (In-Rh@C_60_), these factors determine its adsorption and catalytic properties. Different spin states represent the different electronic configurations, which affect the total energy of the system^49^. The lowest spin energy state indicates the most stable electronic configuration under given conditions and can predict the optimal spin state for adsorption studies^50^. The coordination of In and Rh on C_60_ influences the stability and adsorption of the gas molecules. By examining the different spin states – singlet, triplet, quintet and septet, the most stable electronic configuration can be determined, thus, influencing the modified system’s reactivity towards gas adsorption. The spin states energies were calculated in electronvolts (eV) and presented in **Table** S2. The most stable spin state was the triplet state with an energy of 0 eV, while quintet state has a spin state energy of 0.682 eV, indicating a high stable configuration. The septet and singlet states have the highest spin state energy of 2.557 eV and 1.120 eV, respectively, indicating that transition to these states leads to a less stable configuration. The energetic feasibility of the triplet state suggests that adsorption studies should focus primarily on the spin configurations to capture the most realistic electronic properties of In-Rh@C_60_. However, upon adsorption of NO and NO_2_ gases, the spin polarization effect may consequently shift the system towards a higher spin state (quintet) as a result of unpaired electrons in these gas molecules. These findings show that adsorption and catalytic activity on the modified In-Rh@C_60_ system should be primarily analyzed in the triplet and quintet states, since they dominate the electronic structure and reactivity of the system. If the adsorbed system is stable at higher spin state like quintet, desorption may occur due to the system’s tendency to return back to the triplet stable or ground state. Thus, stronger adsorption, alongside high charge transfer and spin transition, can lead to a spontaneous desorption process.

### Reactivity and stability analysis

**Fig. 4:**
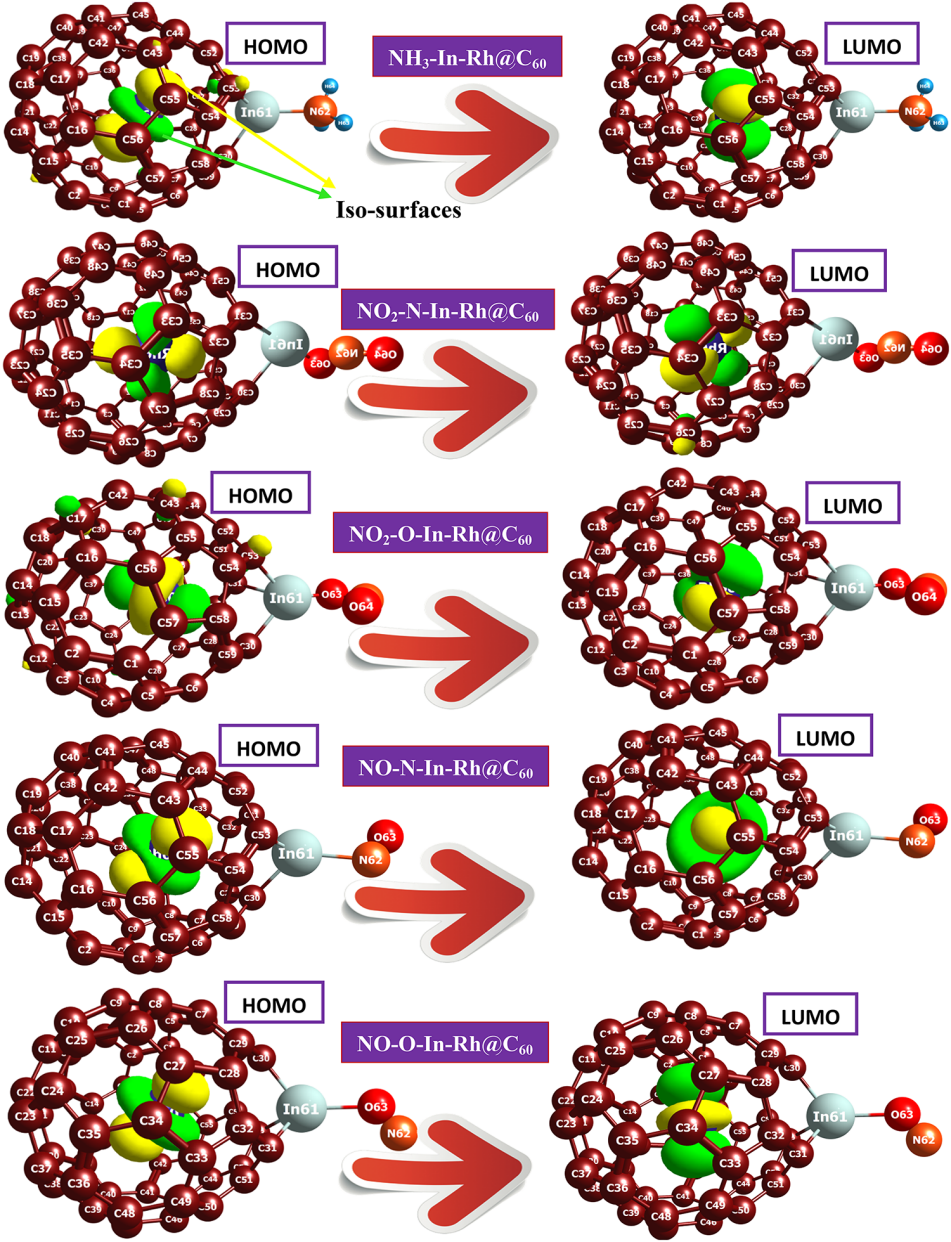
HOMO and LUMO plots of the gas-adsorbed systems.

The calculated values of the lowest unoccupied molecular orbital (LUMO), highest occupied molecular orbital (HOMO), and energy gap for the modified In-Rh@C_60_ system and its derivatives after adsorption are presented in **Table** 2. The study of frontier molecular orbital (FMO) offers valuable insights into the electronic properties, chemical reactivity, and interactions of systems with other molecules. This quantum mechanics technique revolves around the highest occupied molecular orbital and the lowest unoccupied molecular orbital. The energy gap which is the energy difference between the HOMO and LUMO is vital in understanding the properties of material, such as conductivity, electrical, thermal, reactivity, and optical properties^51,52^. Here, the energy gap study, which is the energy difference between the HOMO and LUMO of the system and its derivatives provided an understanding of the crucial properties, such as conductivity, reactivity, and stability. The HOMO and LUMO plots upon the adsorption of gases are depicted in the Fig. 4. The quantum descriptors presented in **Tables** 2 and S3 were computed using the Koopman’s approximation given by Eqs. (2–8).

2 $$\:\mathrm{I}\mathrm{P}={-E}_{\mathrm{H}\mathrm{O}\mathrm{M}\mathrm{O}}$$

3 $$\:EA={-E}_{\mathrm{L}\mathrm{U}\mathrm{M}\mathrm{O}}$$

4 $$\:\mathrm{E}\:\left(\mathrm{e}\mathrm{V}\right)\hspace{0.17em}=\hspace{0.17em}\mathrm{E}\:\left(\mathrm{H}\mathrm{a}\right)\:\times\:\:27.2114$$

5 $$\:\mu\:=-\raisebox{1ex}{$1$}\!\left/\:\!\raisebox{-1ex}{$2$}\right.\left({E}_{\mathrm{H}\mathrm{O}\mathrm{M}\mathrm{O}}+{E}_{\mathrm{L}\mathrm{U}\mathrm{M}\mathrm{O}}\right)$$

6 $$\:H=\raisebox{1ex}{$1$}\!\left/\:\!\raisebox{-1ex}{$2$}\right.\left(IP-EA\right)=\frac{{E}_{\mathrm{L}\mathrm{U}\mathrm{M}\mathrm{O}}-{\mathrm{E}}_{\mathrm{H}\mathrm{O}\mathrm{M}\mathrm{O}}}{2}$$

7 $$\:S=\frac{1}{2{\upeta\:}}=\frac{1}{IP-EA}=\frac{1}{{\mathrm{E}}_{\mathrm{L}\mathrm{U}\mathrm{M}\mathrm{O}}-{\mathrm{E}}_{\mathrm{H}\mathrm{O}\mathrm{M}\mathrm{O}}}$$

The electrophilicity index ($\:\omega$), was calculated using the equation given as:

8 $$\:\omega\:=\frac{{\mu\:}^{2}}{2\mathrm{H}}$$

**Table 2:** Summary of the calculated LUMO, HOMO, energy gap values of In-Rh@C_60_ and gas-adsorbed In-Rh@C_60_ using DFT/PW6B95-D3/GenECP and ωB97X-D/LANL2DZ computational methods.

	Method 1 (PW6B95-D3/GEnECP)			Method 2 (ωB97X-D/LANL2DZ)		
Systems	E_HOMO_	E_LUMO_	E_g_	E_HOMO_	E_LUMO_	E_g_
In-Rh@C_60_	-5.641	-4.187	1.454	-7.662	-3.681	3.980
NH_3_-In-Rh@C_60_	-5.088	-3.569	1.519	-6.419	-1.629	4.790
NO-N-In-Rh@C_60_	-8.945	-8.057	0.888	-7.993	-3.494	4.499
NO-O-In-Rh@C_60_	-8.676	-7.972	0.705	-7.115	-3.963	3.152
NO_2_-N-In-Rh@C_60_	-9.877	-8.722	1.154	-8.539	-3.374	5.166
NO_2_-O-In-Rh@C_60_	-9.591	-8.054	1.537	-8.458	-3.435	5.023

Note: All units are in electronvolts (eV).

The calculated electronic properties of the In-Rh@C_60_ system and its derivatives, determined using two levels of theory, PW6B95-D3/GenECP (Method 1) and ωB97X-D/LANL2DZ (Method 2), reveal distinct trends in HOMO, LUMO, and energy gap values, providing insights into their stability, reactivity, and potential for gas sensing applications. The results from both methods indicate consistent trends for specific systems while highlighting differences in their approximation of electronic properties, which merit detailed analysis.

From the results obtained using Method 1, the energy gap values for the systems ranged from 0.705 eV for NO-O-In-Rh@C_60_ to 1.537 eV for NO_2_-O-In-Rh@C_60_. The notably small energy gap of NO-O-In-Rh@C_60_ suggests higher reactivity due to its lower stability and increased ease of electron excitation. This behavior may be attributed to the destabilizing interaction between NO and the In-Rh@C_60_ framework at the O-site of adsorption. Conversely, NO_2_-O-In-Rh@C_60_, with the largest energy gap, exhibits greater stability and reduced electronic reactivity, possibly due to charge transfer interactions and electronic structural modifications induced by NO_2_ at the O-site^53,54^. The stability trends observed from Method 1 imply that NO_2_-O-In-Rh@C_60_ is the most stable system, whereas NO-O-In-Rh@C_60_ and NO-N-In-Rh@C_60_, with their narrower energy gap values, are the least stable and most reactive.

In comparison, Method 2 yielded energy gap values that are significantly larger across all systems, ranging from 3.152 eV for NO-O-In-Rh@C_60_ to 5.166 eV for NO_2_-N-In-Rh@C_60_. This increase in energy gap values highlights the tendency of Method 2 to overestimate electronic stability of the systems, predicting larger energy gap values compared to Method 1. Interestingly, while the trends for NO-O-In-Rh@C_60_ and NO_2_-O-In-Rh@C_60_ remained consistent with Method 1, Method 2 reversed the ranking of NO_2_-N-In-Rh@C_60_, suggesting it to be the most stable system. This discrepancy highlights the influence of the theoretical approach on the interpretation of electronic properties, with Method 1 potentially offering a more realistic approximation for reactive systems, given its incorporation of dispersion corrections.

The trends observed for HOMO and LUMO energies further substantiate these conclusions. For both methods, NO-O-In-Rh@C_60_ consistently exhibited the least negative HOMO value, indicating its high reactivity and low ionization potential. On the other hand, NO_2_-O-In-Rh@C_60_ showed the most negative LUMO energies, consistent with its high electron affinity and stability. These properties reflect the interplay between the electronic structure of In-Rh@C_60_ and the adsorbed gases, which modulate conductivity, stability, and reactivity based on the nature of the interaction and the adsorption site. The implications of these findings for gas sensing applications are significant. NO_2_-O-In-Rh@C_60_, with its high stability and moderate conductivity, emerges as the optimal candidate for long-term sensing applications where durability is critical. Conversely, NO-O-In-Rh@C_60_, with its superior conductivity, is better suited for dynamic sensing environments, albeit with limitations in long-term stability. The close energy gap values for NH_3_-In-Rh@C_60_ and NO_2_-O-In-Rh@C_60_, as predicted by Method 1, suggest comparable electrical and chemical properties, further supporting their potential utility in stable sensing applications. Generally, the comparison of the two methods highlights the advantages of PW6B95-D3/GENECP (Method 1) in providing a balanced approximation of stability and reactivity, particularly for systems with pronounced electronic interactions. Method 2, while emphasizing stability, may underestimate reactivity and conductivity for certain systems. These differences emphasize the importance of selecting appropriate theoretical methods for studying electronic properties, especially for systems like fullerene. The observed variations in the energy gap values across both methods reinforce the role of adsorption-induced electronic modifications in determining the suitability of these systems for gas sensing applications, offering a pathway for further experimental validation and optimization.

#### Density of States (DOS)

**Fig. 5:**
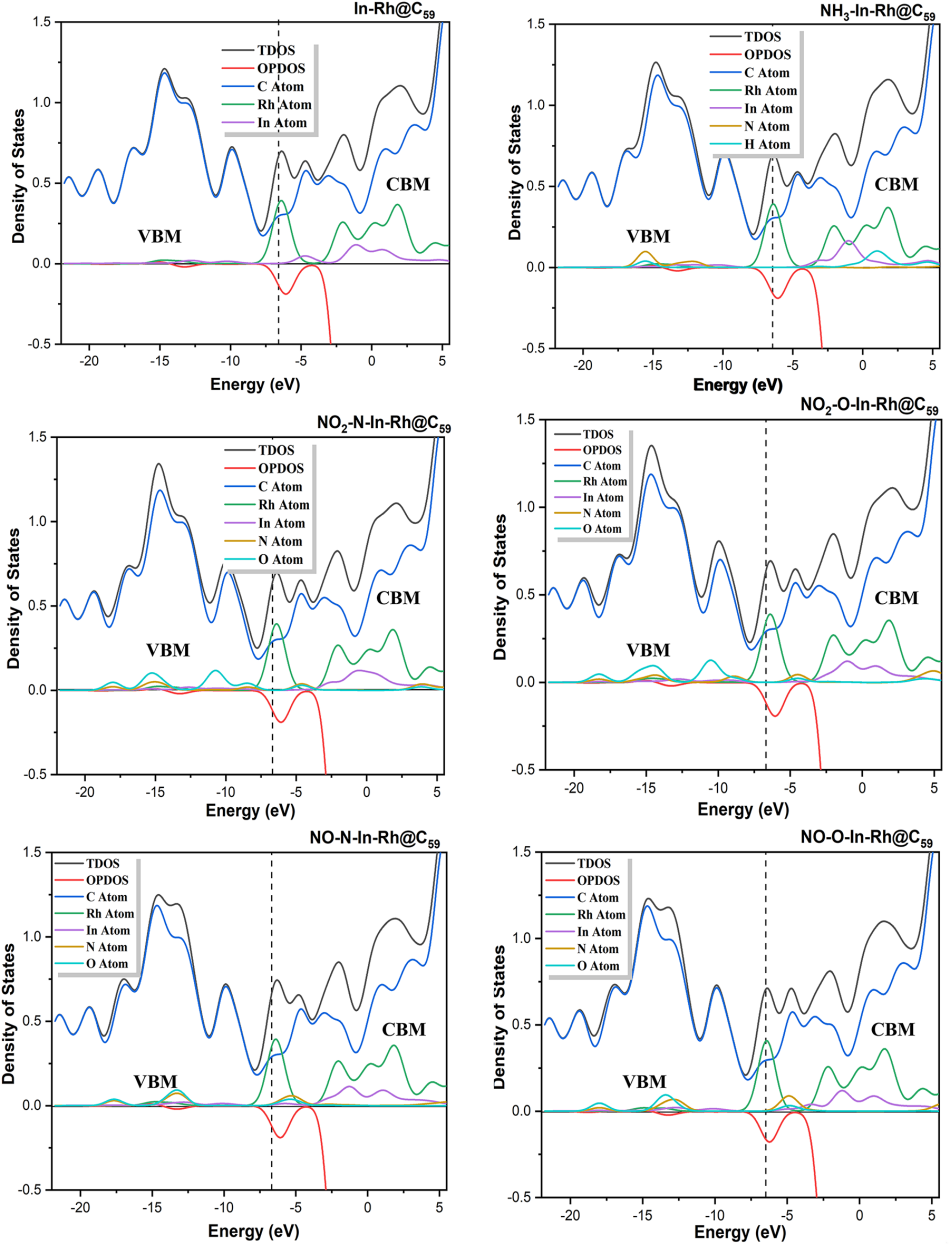
Density of states plots of the modified In-Rh@C_60_ system and gas-adsorbed In-Rh@C_60_ systems calculated at the DFT/PW6B95-D3/GENECP level of theory (Method 1).

**Fig. 6:**
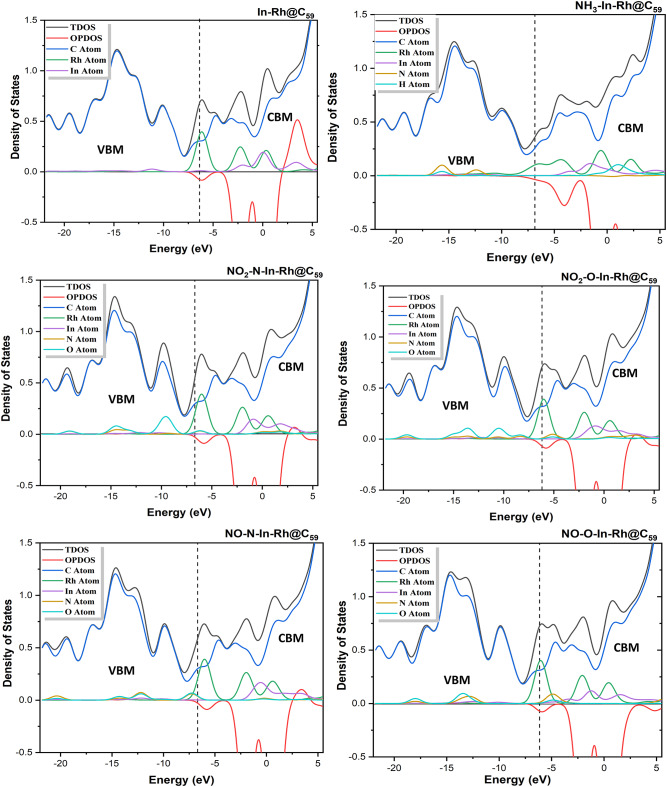
Density of states plots of the modified In-Rh@C_60_ system and the corresponding gas-adsorbed systems calculated at the DFT/ωB97X-D/LANL2DZ level of theory (Method 2).

The Density of States (DOS) serves as a crucial descriptor in evaluating the electronic properties and reactivity of molecular systems. It provides insights into the contributions of atomic orbitals to the electronic structure, particularly in adsorption and charge transfer processes^55^. In this study, the DOS calculations were performed to further elucidate the electronic interactions within the modified fullerene and its functionalized systems upon interaction with NH_3_, NO, and NO_2_ gases. This analysis was conducted using the two DFT methods of this study (PW6B95-D3/GEnECP and ωB97X-D/LANL2DZ) to compare the accuracy of these methods in describing the stability and reactivity of the studied systems. Figures 5 and 6 present the DOS plots, computed using the Multiwfn 3.7 package and visualized with Origin software. For Method 1 (PW6B95-D3/GEnECP), the DOS plots of the systems show a dominant carbon atom peak at the Valence Band Maximum (VBM), reflecting a high electronic contribution. At the Conduction Band Minimum (CBM), carbon and Rh peaks were more pronounced, indicating their strong charge acceptance abilities, with minor contributions from In. Strong In-Rh hybridization at the VBM suggested that the system exhibited good adsorption properties, as Rh near the Fermi level could participate in charge transfer. This observation is consistent with studies on transition metal-functionalized silicon carbide nanotubes (TM@SiCNTs), where similar charge transfer interactions were reported for cobalt, Rh, and iridium in trichloromethane gas adsorption^56^. It is worth noting that carbon exhibited the highest peak at the VBM and CBM indicating its ability to donate and accept charges, thus contributing to the stability of the system. Upon interacting with NH_3_ molecule, a shift in the peaks was observed, as an overlap (hybridization) of the Rh, In, N and O peaks was observed at the VBM with a slight elevation of N peak, indicating its tendency to donate charges and promote electron localization. At the CBM, Rh exhibited the highest peak, followed by In revealing their tendency to accept charges. During the interaction of the modified In-Rh@C_60_ system with NO_2_ at both sites of adsorption (N and O), the O atom peak was observed to be slightly elevated in the VBM region, indicating a potential for charge transfer, while an overlap was observed between N, Rh and In atoms. This behavior agrees with previous findings on metal-doped fullerenes, where gallium and In dopants introduced new electronic states near the Fermi level, enhancing charge transfer and adsorption^57^. In the CBM region, Rh and In peaks were more pronounced, while an overlap between N and O peaks was observed, indicating high reactivity and catalytic activity. Upon interaction with NO gas at N and O, peaks were observed to overlap each other and slightly elevated in the VBM region, depicting a measure of charge transfer. An overlap between Rh, In, N, O was observed near the Fermi level. However, in the CBM region, Rh and In were observed to possess the highest peak, with N & O peaks overlapping each other. This indicates stronger conductivity especially for sensing and adsorbing NO gas.

For Method 2 (ωB97X-D/LANL2DZ), the carbon peak was equally observed to have the highest peaks at both VBM and CBM. The DOS plots of the systems reveal hybridization between Rh and In peaks in the VBM region, while Rh and In peaks were elevated in the CBM region, indicating high charge acceptance. Upon adsorption of NH_3_, all the peaks of each fragment overlapped, with a slight elevation of N & O peaks in the VBM region. Meanwhile, Rh, In, and H peaks were elevated in the CBM region. Upon adsorption of NO_2_ gas at the two sites of adsorption, the O atom peak was observed to be slightly elevated, while hybridization was observed between Rh, In, and N peaks in the VBM region. On the other hand, in the CBM region, In and Rh had high peaks while N and O overlapped. Upon adsorption of NO gas at both sites of adsorption, all the atom fragments overlapped in the VBM region, accompanied by a slight elevation of N and O peaks near the fermi level. In the CBM region, Rh and In exhibited the highest peaks, while an overlap between N and O was observed.

#### Electron localization function (ELF)

**Fig. 7:**
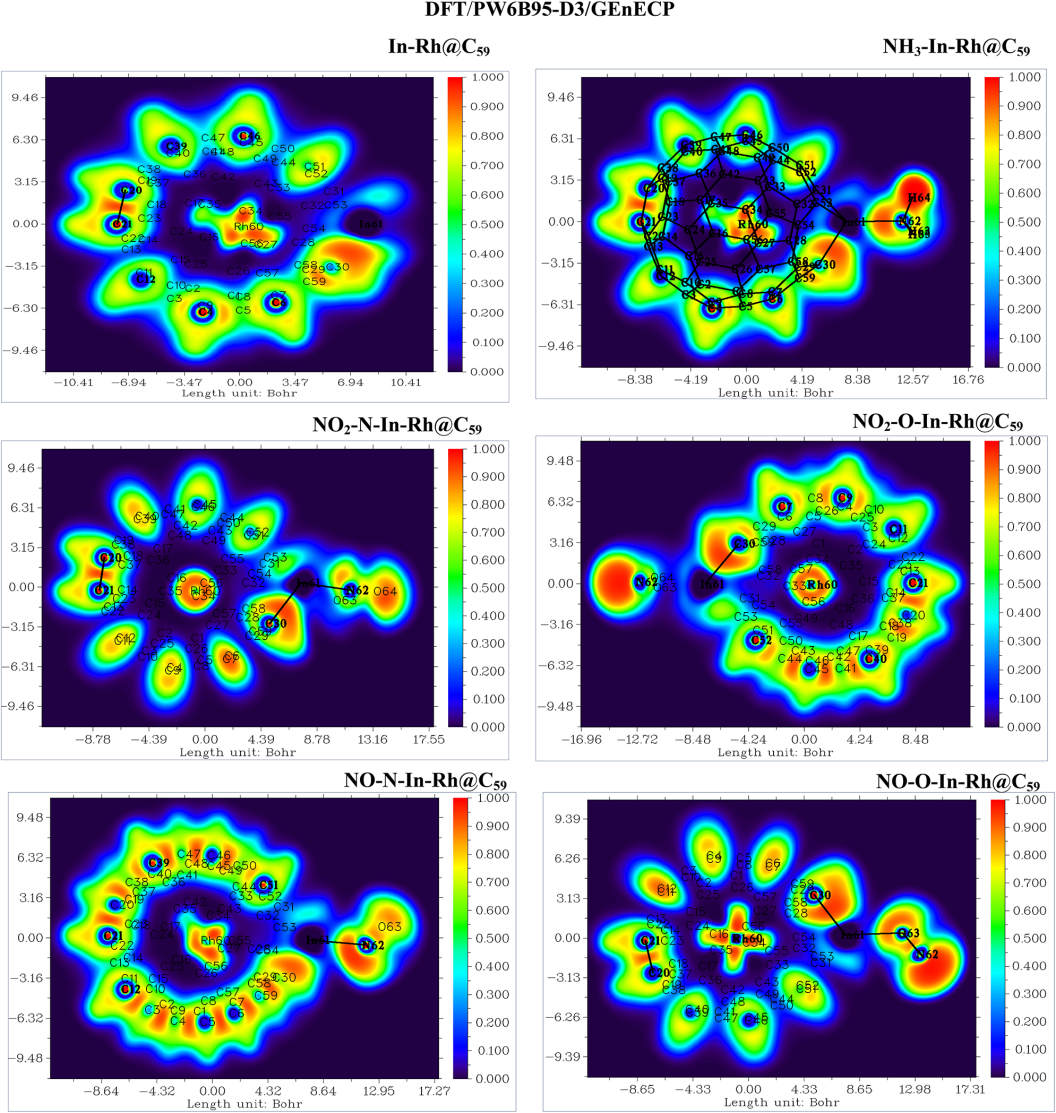
Electron Localization Function (ELF) analysis of the modified In-Rh@C₅₉ system and the corresponding gas-adsorbed systems calculated at the DFT/PW6B95-D3/GENECP level of theory. The ELF plots illustrate electron density distribution, highlighting bond localization and charge delocalization at the adsorption sites.

**Fig. 8:**
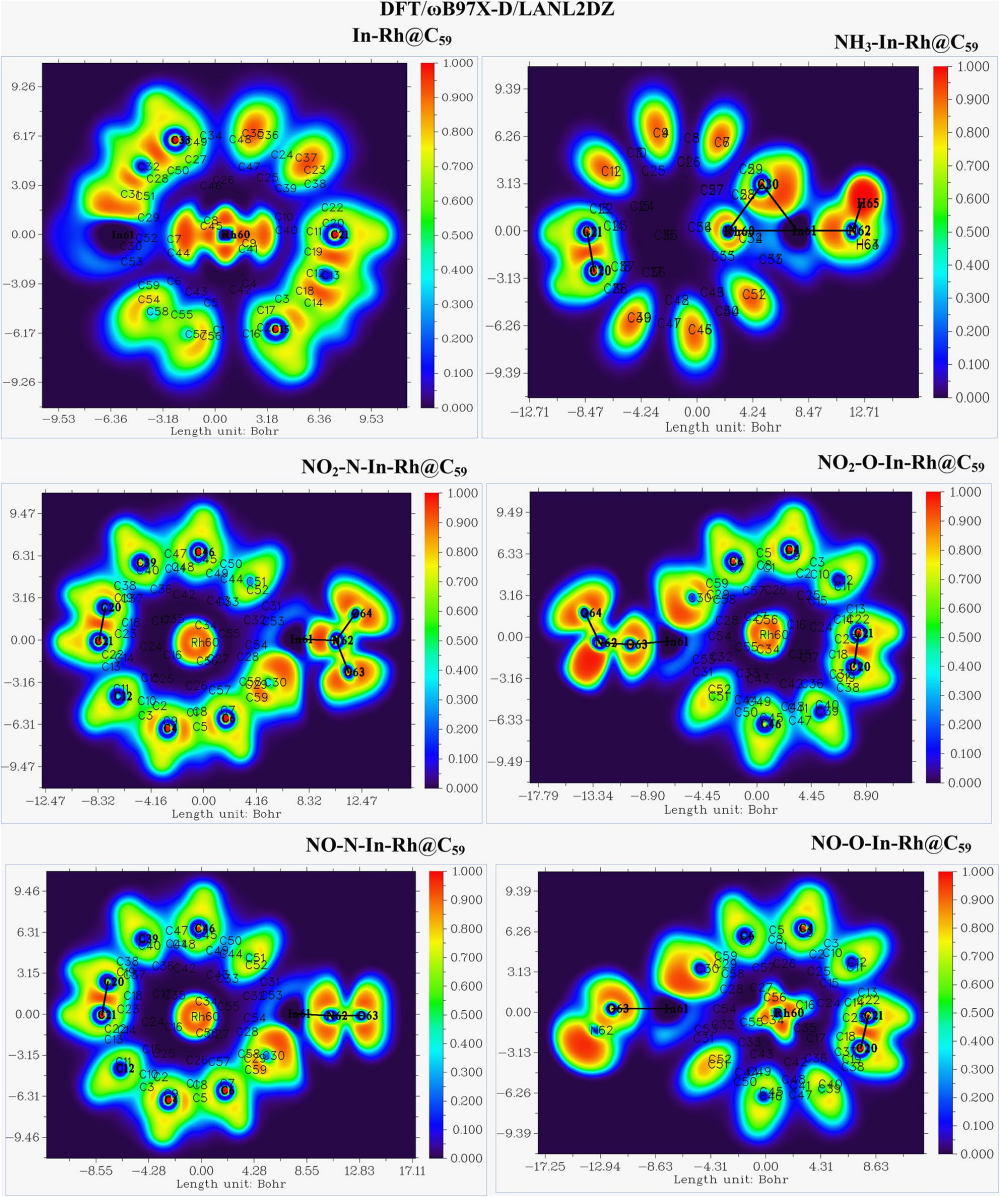
Electron localization function (ELF) analysis of the modified In-Rh@C_59_ system and the corresponding gas-adsorbed systems calculated at the DFT/ωB97X-D/LANL2DZ level of theory. The ELF plots illustrate electron density distribution, highlighting bond localization and charge delocalization at the adsorption sites.

The Electron Localization Function (ELF) analysis provides crucial insights into electron density distribution and bonding characteristics of the adsorbed gases with In-Rh@C_59_ system (Figs. 7 and 8). According to Akman et al.., 2023, ELF values less than 0.5 signify delocalization of electrons, while ELF values greater than 0.5 indicate localization of electrons^58^. The ELF scale for the DFT/PW6B95-D3/GEnECP and DFT/ωB97X-D/LANL2DZ methods ranges from 0 (blue regions, representing delocalized electrons) to 1 (red regions, representing highly localized electrons or strong covalent bonding), while the intermediate values indicate polar or shared interactions. The ELF for both methods shows similar charge localization trends, confirming a consistent charge transfer mechanism. Generally, from the ELF-mapped plot of the first DFT method, the Rh atom exhibits moderate electron localization (0.5 - 0.8) suggesting a partially covalent bond with the C_59_ system. The In atom, with a blue color, signifies delocalization, which implies the transfer of charges from the doped atom to the fullerene system. The carbon-to-carbon interactions of the fullerene framework display strong electron localization (0.8 - 1.0), which reflects its sp^2^-hybridized center. Some regions of the fullerene cage show lower localization (0.4 - 0.6), which signifies modified π-electron delocalization due to Rh and In doping. The presence of the adsorbed molecules alters the ELF-mapped plot. ELF near the adsorbed molecule ranges from 0.4 to 0.9, signifying partial covalent bonding and electron transfer. This charge redistribution enhances the adsorption activity of the systems. Partial electron localization near the system’s adsorption sites confirms active charge transfer, while the presence of strong covalent bonding within the fullerene and weaker interactions at the adsorption site (dopants) ensures stability and reactivity.

### Adsorption of gases on the modified In-Rh@C_60_ fullerene system

Previously, the adsorption of NO and NO_2_ gas molecules has been studied theoretically to determine the intricate properties of the bare C_60_ fullerene. In line with this, many reports have been made regarding the adsorbing potency of the bare C_60_ fullerene, as well as their functionizedsystems^59–61^. In this study, the C_60_ fullerene was structurally modified by encapsulating the Rh atom within its hollow cage-like structure and doping it through the substitution of one carbon atom with an In atom. The newly designed Rh-encapsulated In-doped C_60_ fullerene was then used in the adsorption of NH_3_, NO, and NO_2_ by considering two sites of adsorption in the case of NO and NO_2_. The most stable adsorption configurations were identified, and the results obtained using Eq. (1) are presented in **Tables** 3 and 4. In all cases, chemisorption was obtained for the labeled systems, showing that the designed system is a promising adsorbent material for NH_3_, NO_2_, and NO_2_ gas molecules. In the following sections, we examine the adsorption of these gases individually to evaluate the influence of sites on the adsorption.

#### NH_3_ adsorption on the modified In-Rh@C_60_ system

Considering the N-site only, NH_3_ gas was adsorbed on the modified In-Rh@C_60_ system, with a high adsorption energy of -12.11 eV. This energy obtained depicts a chemisorption phenomenon and indicates that the NH_3_ gas molecules were strongly adsorbed on the modified system of In-Rh@C_60_. The relatively greater energy shows that among the studied adsorbates, NH_3_ will be strongly adsorbed on the modified system compared to the adsorption of the NO gas molecules.

#### NO adsorption on the modified In-Rh@C_60_ system

Due to the presence of N and O atoms of the NO gas molecules, the N- and O-sites of adsorption were considered during the adsorption process. The adsorption energies of -8.397 and -7.690 eV corresponding to the N- and O-sites of adsorption were obtained upon adsorption. Both values indicate strong adsorption of NO gas on the In-Rh@C_60_ system. Since the adsorption at the N-site shows greater energy than at the O-site, the N-site exhibited relatively stronger adsorption.

#### NO_2_ adsorption on the modified In-Rh@C_60_ system

In the adsorption of NO_2_ on the modified In-Rh@C_60_ system, the adsorption energy falls within a close range of -13.49 eV to -12.15 eV, where the highest energy is attributed to the adsorption at the O-site. Since a more negative adsorption energy indicates stronger adsorption^62^, the adsorption configuration at the O-site exhibits the most favorable adsorption compared to the N-site of adsorption. In all cases, the strongest adsorption was seen in NO_2_-O-In-Rh@C_60_. Generally, the adsorption follows this trend: NO-N-In-Rh@C_60_ < NO-O-In-Rh@C_60_ < NO_2_-N-In-Rh@C_60_ < NO_2_-O-In-Rh@C_60_, representing an increasing order of adsorption strength.

Compared to Method 2 (DFT/ωB97XD/LANL2DZ), the adsorption energies are higher, which signifies strong interactions between the gases and the modified system. However, the limitation is the desorption process might be difficult due to the strong bonds between the adsorbate and the adsorbent. Thus, the adsorption energy of Method 2 is preferable based on the reactivity studies, as it indicates higher chemical reactivity and a more favorable adsorption-desorption process.

**Table 3:** Adsorption energies of the gas-adsorbed systems calculated at the DFT/PW6B95-D3/GenECP level of theory.

Systems	E_systems_/Ha	E_adsorbent_/Ha	E_adsorbate_	E_ads_ (Ha)	E_ads_ (eV)
NH_3_-In-Rh@C_60_	-2417.612985	-2360.979556	-56.188528	-0.4449	-12.11
NO_2_-N-In-Rh@C_60_	-2565.917197	-2360.979556	-204.491072	-0.4466	-12.15
NO_2_-O-In-Rh@C_60_	-2565.966476	-2360.979556	-204.491072	-0.4956	-13.49
NO-N-In-Rh@C_60_	-2490.678128	-2360.979556	-129.389988	-0.3086	-8.397
NO-O-In-Rh@C_60_	-2490.652131	-2360.979556	-129.389988	-0.2826	-7.690

**Table 4:** Adsorption energies of the gas-adsorbed systems calculated at the DFT/ωB97XD/LANL2DZ level of theory.

Systems	E_systems_/Ha	E_adsorbent_/Ha	E_adsorbate_	E_ads_ (Ha)	E_ads_ (eV)
NH_3_-In-Rh@C_60_	-2414.76	-2357.12	-56.5295	-1.1024	-29.9979
NO_2_-N-In-Rh@C_60_	-2487.9	-2357.12	-129.639	-1.13617	-30.9169
NO_2_-O-In-Rh@C_60_	-2487.95	-2357.12	-129.639	-1.19267	-32.4543
NO-N-In-Rh@C_60_	-2563.11	-2357.12	-204.963	-1.01919	-27.7335
NO-O-In-Rh@C_60_	-2563.12	-2357.12	-204.963	-1.03627	-28.1984

#### Thermodynamic analysis

The thermodynamics properties of the studied systems were calculated at the DFT/ωB97XD/LANL2DZ level of theory to further elucidate their adsorption properties, as well as the strength and stability of interactions between the gases (NH_3_, NO, and NO_2_) and the modified system (In-Rh@C_60_). The calculations for all systems were performed at an absolute temperature of 298.15 K (25^0^ C) and standard pressure of 1 atm, as presented in **Table S4**. The spontaneity and feasibility of the interactions between In-Rh@C_60_ and NH_3,_ NO_2_, and NO gases can be determined using thermodynamic parameters, thereby confirming the potential of the modified system for detecting and sensing of the pollutant gases^63^. Thermodynamics parameters such as enthalpy change (ΔH^0^) and Gibbs free energy change (ΔG^0^) are key indicators for determining the thermal stability and spontaneity of the interactions between In-Rh@C_60_ and NH_3_, NO_2_, and NO gases^64^. The electronic energy (Ɛ_0_), zero-point energy correction (Ɛ_ZPE), thermal correction to energy (E_tot_), thermal correction to enthalpy (H_corr_), thermal correction to free energy (G_corr_), Ɛ_0_ + zero-point Energy, Ɛ_0_ + thermal Energy correction, Ɛ_0_ + thermal enthalpy correction, Ɛ_0_ + thermal free energy correction, change in Gibbs free energy (ΔG^0^), and change in enthalpy (ΔH^0^) are all presented in **Table S4**. The change in Gibbs free energy (ΔG^0^) and change in enthalpy (ΔH^0^) associated with the adsorption of the gas molecules onto the modified system were calculated using the following mathematical expression:

3 $$\:{\varDelta\:H}_{ads}={H}_{system}-({H}_{gas}+{H}_{surface})$$

4 $$\:{\varDelta\:G}_{ads}={G}_{system}-({G}_{gas}+{G}_{surface})$$

Where *H*_*system*_ is the enthalpy of the gas-adsorbed system, *H*_gas_ is the enthalpy of the individual gas molecules (NH_3_, NO_2_, and NO), *H*_surface_ is the enthalpy of the modified system, *G*_*system*_ is the Gibbs free energy of the gas-adsorbed system, G_gas_ is the Gibbs free energy of the the individual gas molecules (NH_3_, NO_2_, and NO), and *G*_surface_ is the Gibbs free energy of the modified system. The electronic energy (Ɛ_0_) shows that NO_2_-O-In-Rh@C_60_ is the most stable, with the highest value of -69746.1 eV. In contrast, In-Rh@C_60_ has the least Ɛ_0_ value, indicating an increase in stability upon interaction with the gas molecules. NH_3_-In-Rh@C_60_ exhibits the highest E_tot_ and H_corr_, suggesting higher thermal energy involvement, which may influence its practical stability. NO-O-In-Rh@C_60_ shows the least G_corr_ value of 8.9852 eV, indicating a thermodynamic preference for adsorption-based applications. The calculated ΔG^0^ reveals that NH_3_-In-Rh@C_60_ exhibits the highest positive value, indicating an endothermic process, non-spontaneous and thermodynamically unfavorable adsorption process. In contrast, NO-N-In-Rh@C_60_ and NO-O-In-Rh@C_60_, with ΔG^0^ values of -0.0716 and -0.1208 eV, respectively, indicate a spontaneous adsorption process. Similarly, ΔH^0^ analysis reveals that NH_3_-In-Rh@C_60_ and NO_2_-O-In-Rh@C_60_ undergo an endothermic adsorption process with enthalpy values of 2.2176 and 0.0936 eV, respectively. However, NO-N-In-Rh@C_60_ and NO-O-In-Rh@C_60_ exhibit an exothermic process, with enthalpy values of -0.0523 and -0.0046 eV, respectively, which implies the spontaneity of adsorption process. These results suggest that In-Rh@C_60_ has a greater tendency to detect and sense NO gas molecules under standard temperature and standard pressure (298.15 K, 1 atm).

### Visual studies

#### Non-covalent interaction studies

**Fig. 9:**
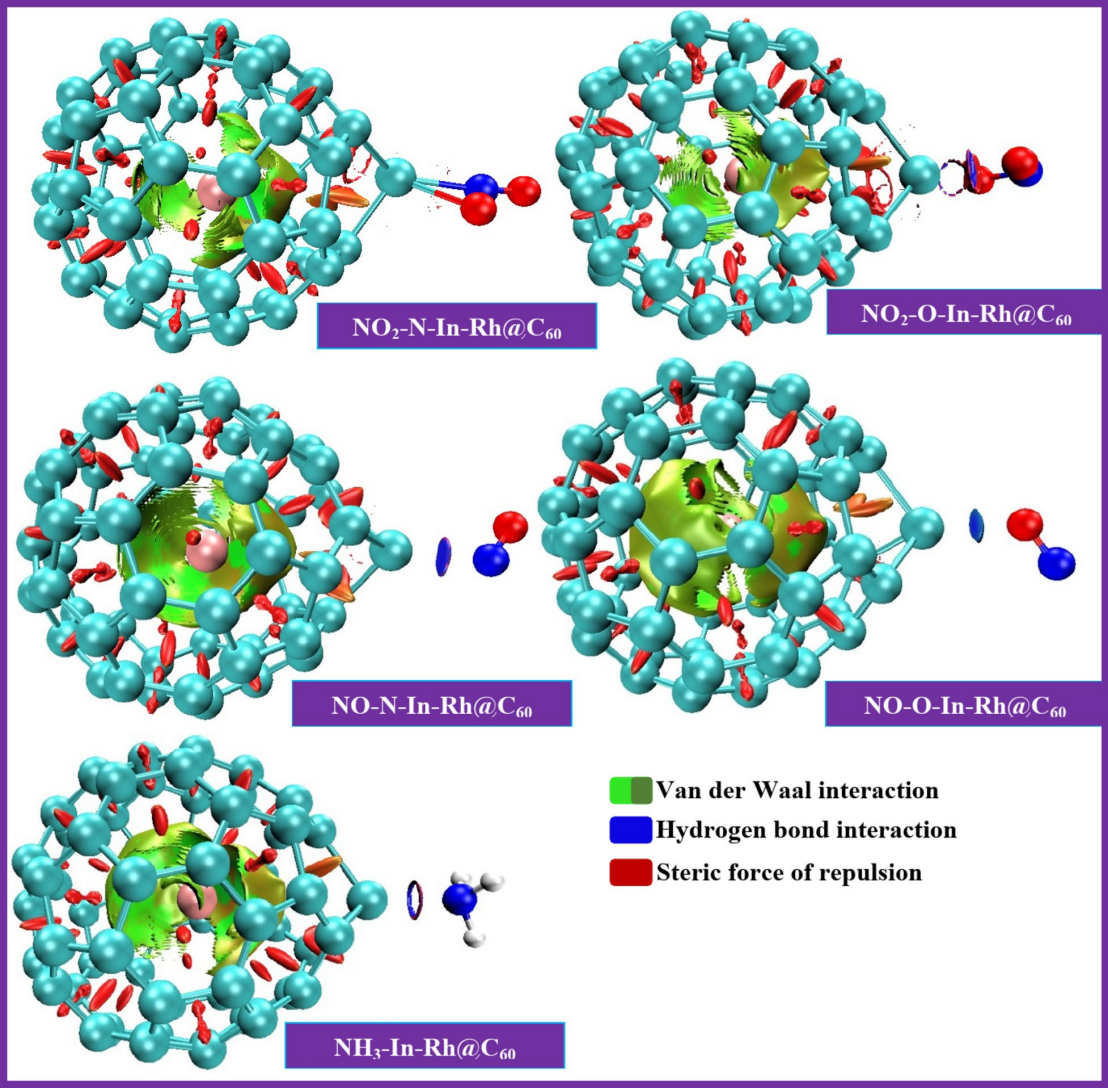
3D plots of the non-covalent interactions of the systems.

Non-Covalent Interaction (NCI) studies as applied to this investigation between adsorbates and adsorbents give relevant insights into the nature of weak intermolecular forces governing adsorption phenomena^65^. They offer a broad basis for understanding non-covalent interactions such as van der Waals forces, hydrogen bonding, π-π stacking, and electrostatic interactions, which play crucial roles in adsorbate-adsorbent interactions^66^. In this context, we utilized DFT to analyze the electron density and energy landscapes associated with adsorption processes. By visualizing the spatial distribution of non-covalent interactions, we identified regions of attractive and repulsive forces between adsorbates and adsorbents, thereby providing vital insights into the binding geometries and adsorption energetics^67^. It has been reported that this analysis allows for the quantification of interaction energies, enabling the prediction of adsorption strengths and selectivity for different adsorbate-adsorbent pairs^68^. Based on our NCI results depicted in Fig. 9, all the studied systems displayed large green patches in the mesoporous of the fullerene C_60_ material. This could be influenced by the encapsulation of Rh, a transition metal exhibiting nucleophilic behavior. Thus, the green color depicts the van der Waals force of interactions, thereby contributing to the system’s stability behavior. Whereas the red color captured in the intramolecular bonding of the systems depicts the steric force of repulsion. The two forces enhance the system’s capability to adsorb gases from the environment. Conversely, NO_2_-O-In-Rh@C_60_ (adsorbent binding to the O-site of NO_2_ gas) was observed with steric and strong attraction (H-bonding) in its intermolecular regions, implying greater adsorption compared to NO_2_-N-In-Rh@C_60_ (adsorbent binding to the N-site of NO_2_ gas) which did not display any electrostatic force in its intermolecular bonding. Examining the adsorption of NO gas, at both the N- and O-sites, the intermolecular regions were notable with blue patches, which depicted strong attraction or H-bonding. This indicates strong interaction at any site of the NO gas molecules, offering strong hydrogen bonding between the studied modified system and the adsorbate (gas). A similar character was also observed when the studied system interacted with NH_3_. Hence, the insights gleaned from this study can play a crucial role in designing and optimizing materials for sensor applications, given the investigation of diverse electrostatic forces. This highlights the significance of this work in advancing sensor technology.

#### QTAIM analysis

The Quantum Theory of Atoms in Molecules (QTAIM) is well known for investigating interactions between adsorbates and adsorbents at the atomic and molecular levels^67–69^. This theoretical framework enables the detailed analysis of electron density distributions and the topological features of chemical bonds and presents an essential understanding of adsorption. Therefore, by conducting QTAIM analysis, we have defined the nature and strength of intermolecular and intramolecular interactions within the systems resulting in the clarification of the mechanisms underlying the adsorption process. Herein, crucial insights were gained by identifying critical points in the electron density distribution, such as bond critical points (BCPs), which characterize the regions of significant electron density accumulation between atoms^68^. These BCPs provide valuable information about the nature of chemical bonding, including covalent, ionic, or van der Waals interactions, thus, providing insights into the stability and geometry of adsorbate-adsorbent systems. Furthermore, this analysis facilitates the investigation of charge transfer phenomena and the redistribution of electron density upon adsorption. The calculated results presented in **Table** S1 and **Figure** S1 demonstrated a positive value of the Laplacian of charge density, as observed in the intermolecular and intramolecular regions. This indicates a decrease in the values of charge density between the modified system and gas molecules, thus presenting a typical characteristic of closed-shell interactions. The Laplacian of charge density value of ∇^2^ ρ(r) at 85,73, and 62 critical points (CP) of the NO_2_-N-In-Rh@C_60_ system was relatively higher (0.4516 a.u, 0.5833 a.u, and 0.3699 a.u) as compared to the other systems. Furthermore, electron localization and delocalization within the systems were investigated through the electronic localization function (ELF), where an ELF value less than zero indicates high delocalization, while a value greater than zero is indicative of localization regions^70,71^. All the studied systems exhibited delocalization of electrons at most of their critical points (see **Table** S1). However, the least value of ELF (0.2921 a.u) was observed at the bond S_93_-N_107_ and 73 critical points, as well as the In_61_-N_63_ bond length for the NO_2_-N-In-Rh@C_60_ system, indicating electronic mobilization between the atom of the gas and the modified system. Additionally, more characteristics of the interactions can be accounted for by the interaction between electrons and nuclei of the systems, and the value of potential energy density V(r) can be utilized for this prediction, as higher values indicate significantly strong interactions^72^. Surprisingly, the V(r) values recorded from some critical points were negative across all the systems, indicating a weak force of interactions. This analysis reveals key findings on adsorbate-adsorbent interactions, enhancing the understanding of their electronic structure and bonding characteristics in sensor materials.

### Sensor mechanisms

Determining how the newly tailored material can be used in real-time applications, such as in the design of a sensor device, can be achieved through sensing mechanisms. Here, the work function, fraction of electron transfer, back-donation, and conductivity were utilized to determine the material’s suitability for sensing applications.

#### Change in work function

The percentage change in the work function (%Δϕ) was computed to examine the percentage increase of the work function during the adsorption process. Before adsorption, the newly engineered materials possessed a work function of 4.914 eV, and this was found to increase upon adsorption to 9.299 (89.24%), 8.823 (79.55%), 8.501 (73.00%), and 8.324 eV (69.39%) for NO_2_-N-In-Rh@C_60_, NO_2_-O-In-Rh@C_60_, NO-N-In-Rh@C_60_, and NO-O-In-Rh@C_60_ respectively (see **Table** 5). However, a slight decrease in work function was observed for NH_3_-In-Rh@C_60_ with a work function and percentage increase of 4.329 (-11.91%). Several studies have shown that an increase in the work function of a system is a desirable attribute for performing the required function, and in this case, the said attribute is the adsorbing tendency.

#### Charge transfer

The natural charge on the adsorbate and adsorbent were computed to investigate the mechanism of charge transfer (Qt). Theoretically, Qt can be calculated as the difference between the charge on the adsorbate and that on the adsorbent, as shown in Eq. (9)^73^:

9 $$\:{Q}_{t}={Q}_{adsorbent}-{Q}_{adsorbate}$$

The charge transfer from the adsorbent to the adsorbate was observed for all the systems, as indicated by their positive Qt values (see **Table** 5). This implies that the transfer of electrons has occured, confirming the interaction between the gas molecules and the In-Rh@C_60_ system.

#### Electronic conductivity

Mathematically, the electrical conductivity can be related to the energy gap via Eq. (10) as follows:

10 $$\:{\upsigma\:}=\mathrm{A}{T}^{2/3}{e}^{\left({E}_{g}/2KT\right)}$$

where electrical conductivity (σ), Boltzmann constant (k), temperature (T), energy gap (E_g_), and pre-exponential factor (A) are denoted accordingly. Based on this equation, a high value of E_g_ implies high conductivity^74^. The energy gap values are within a relatively small range of 0.705 to 1.537 eV, showing a considerable and acceptable level of conductivity, which is in agreement with previously reported findings^75,76^. In most cases, the energy gap is reduced upon adsorption, and this has been expressed in the percentage change in the energy gap summarized in **Table** 5. The highest change in the energy gap of -0.515 (51.51%) and -0.389 (38.93%) is associated with the adsorption of NO gas at the O- and N-sites, respectively. These results reveal the contribution of the modified In-Rh@C_60_ fullerene system in demonstrating desirable adsorption properties while reducing the energy gap upon adsorption.

#### The FET and Back-donation mechanisms

The fraction of electron transfer (FET) can be obtained when electrons move from the tailored system to the gases. Similarly, the mobility of electrons back from the gases to the modified In-Rh@C_60_ system can be illustrated using the electrical back donation. A material suitable for sensing gases often has a negative back donation (ΔE < 0) with positive global hardness (η > 0). Equations (11) and (12) present the mathematical relationships used to calculate the FET and back-donation based on the Pearson theory^77^.

11 $$\:\varDelta\:N=\frac{{{\upchi\:}}_{\boldsymbol{i}\boldsymbol{s}\boldsymbol{o}\boldsymbol{l}\boldsymbol{a}\boldsymbol{t}\boldsymbol{e}\boldsymbol{d}}-\:{{\upchi\:}}_{\boldsymbol{s}\boldsymbol{y}\boldsymbol{s}\boldsymbol{t}\boldsymbol{e}\boldsymbol{m}}}{2\:\left({\eta\:}_{isolated\:}-\:{\eta\:}_{system}\right)}$$

12 $$\:{\varDelta\:E}_{Back\:donation}=-\frac{\mathbf{\eta}}{4}$$

In all cases, the back donation values are negative (ΔE < 0), with the highest negative values of -0.1923, -0.1443, and -0.1110 corresponding to NO-O-In-Rh@C_60_, NO-N-In-Rh@C_60_, and NO_2_-N-In-Rh@C_60_. The least negative ΔE value of -0.08800 is attributed to NO_2_-O-In-Rh@C_60_. These results correlate with those obtained in the adsorption energy and are consistent with the observed order of adsorption strength.

**Table 5:** Sensor mechanism parameters for the systems calculated at the DFT/PW6B95-D3/GenECP level of theory.

Systems	D	ϕ(eV)	$\:\mathbf{\%}\varDelta\:\boldsymbol{\upvarphi\:}$	Q_t_ (e)	ΔN	ΔE_BD_	$\:\varDelta\:{\mathbf{E}}_{\mathbf{g}}$	$\:\mathbf{\%}\varDelta\:{\mathbf{E}}_{\mathbf{g}}$
NH_3_-In-Rh@C_60_	13.338	4.329	-11.91	0.538	0.1525	-0.1900	0.0447	4.4704
NO-N-In-Rh@C_60_	2.793	8.501	73.00	1.658	-1.8982	-0.1443	-0.389	-38.927
NO-O-In-Rh@C_60_	4.834	8.324	69.39	2.187	-2.4821	-0.1923	-0.515	-51.513
NO_2_-N-In-Rh@C_60_	9.237	9.299	89.24	1.517	-1.0660	-0.1110	-0.206	-20.633
NO_2_-O-In-Rh@C_60_	3.886	8.823	79.55	1.903	0.2885	-0.0880	0.0571	5.7084

Note: D = polarity. The units of E_ad_, ΔE_g_, and ΔE_back−donation_ (BD) are in electron volts (eV). Qt is expressed in electrons (*e*), and %ΔE_g_ is given in percentage (%).

## Conclusions

The design and modification of new adsorbent material for the adsorption of toxic gas pollutants, such as NH_3_, NO, and NO_2_ have been successfully carried out in this study. With a special focus on the sites of adsorption for NO and NO_2_ adsorption, the N- and O-sites have been carefully examined to determine the most favorable site of adsorption. The DFT approach was adopted and calculations were performed using the DFT/PW6B95-D3/GenECP and ωB97X-D/LANL2DZ computational methods. Comprehensive computational analyses were conducted, including geometry optimization, adsorption energy, electronic properties (FMO analysis), visual studies (AIM and RDG analyses), and sensor mechanism investigation, to uncover useful insights into the intricacies of gas adsorption on the newly engineered system. Hence, the following scientific conclusions are drawn:

1. Adsorption affects the morphology of the cage-like structure of the In-Rh@C_60_ system, resulting in system stretching upon gas adsorption.
2. The energy gap reduced upon adsorption, indicating enhanced reactivity of the system. The energy gap values calculated using Method 1 (PW6B95-D3/GenECP) were found to be within a range of 0.705 eV to 1.537 eV, while Method 2 (ωB97X-D/LANL2DZ) yielded higher energy gap values ranging from 3.152 eV to 5.166 eV, reflecting a tendency to overestimate electronic stability. However, the overall trends in stability and reactivity across the studied adsorption sites remained consistent between both methods.
3. The adsorption phenomena observed across all systems are best described as chemisorption, with the adsorption energies ranging from -13.49 eV to -8.397 eV.
4. Non-covalent interaction (NCI) analysis revealed van der Waals interactions as a key stabilizing force in all the systems, depicted by the green coloring in the visual maps. Notably, NO_2_-O-In-Rh@C_60_ was observed with steric and strong attraction (H-bonding) in its intermolecular regions, implying stronger adsorption compared to NO_2_-N-In-Rh@C_60_, which lacked electrostatic interactions on its intermolecular bonding. Additionally, the blue patches observed in the intermolecular region during the adsorption of NO gas, at both N- and O-sites, depict strong attraction or H-bonding.
5. All the studied systems exhibited delocalization of electrons at most of their critical points and the negative V(r) values recorded at some critical points across all the systems confirmed the presence of weak force of interactions.
6. The charge transfer from the adsorbent to the adsorbate was observed for all the systems, as reflected in their positive Qt values. In all cases, the back donation values are negative (ΔE < 0). Additionally, the highest changes in the energy gap, -0.515 eV (51.51%) and -0.389 eV (38.93%), are associated with the adsorption of NO gas at the O- and N-sites, respectively. These results reveal the role of the In-Rh@C_60_ fullerene system in achieving favorable adsorption behavior while reducing the energy gap upon gas adsorption.

Overall, this study demonstrates that the newly engineered In-Rh@C_60_ system is a promising material for use as a sensitive and high-performance gas sensor capable of detecting hazardous gases such as NH_3_, NO, and NO_2_. Thus, this material shows strong potential for applications in environmental monitoring, industrial safety, emissions control, and air quality assessment.

## Electronic supplementary material

Below is the link to the electronic supplementary material.

Supplementary Material 1

## Data Availability

All data are contained within the manuscript and its supporting information document (ESI). The available software programs employed for the computational studies include Gaussian16, GuassView6.0.16, and CYLview Visualization and Analysis Software. Other software codes include Chemcraft, Multiwfn Analyzer, and the Visual Molecular Dynamic (VMD) Program freely available at http://sobereva.com/multiwfn/ and https://www.ks.uiuc.edu/Research/vmd/ respectively.
