# Supplementary material for: Engineering rhodium encapsulated indium doped fullerene for NH3, NO, and NO2 sensing
Source: Sci Rep. 2025 Dec 24;15:45676. doi: 10.1038/s41598-025-93796-7 (PMC12753856; doi:10.1038/s41598-025-93796-7)
Supplement: Supplementary file 1 — Supplementary Material 1 [file 41598_2025_93796_MOESM1_ESM.docx]

**Table S1: QTAIM results of the investigated systems, showing critical point (CP), density of all electrons ρ(r), Laplacian of the charge density ∇2 ρ(r), kinetic energy density G(r), Hamiltonian kinetic energy K(r), potential energy density V(r), energy density H(r), electron localization function (ELF), (LOL), ellipticity of electron density, localized orbital locator (𝜀), and eigenvalue of hessian(λ_2_)**

| Systems | BONDS | CP | ρ(r) | ∇2ρ(r) | V(r) | G(r) | K(r) | H(r) | ELF | LOL | 𝜀 | λ_2_ |
| --- | --- | --- | --- | --- | --- | --- | --- | --- | --- | --- | --- | --- |
| NH3-In-Rh@C60 | In61-N62 | 84 | 0.6217 | 0.3023 | -0.2312 | 0.2360 | -0.4815 | 0.4815 | 0.8386 | 0.3692 | 0.0011 | -0.8109 |
|  | In61-C3 | 63 | 0.1107 | 0.4126 | -0.6649 | 0.4019 | 0.2629 | -0.2629 | 0.9109 | 0.4851 | 0.0655 | 0.7069 |
|  | In61-C53 | 33 | 0.2745 | -0.7123 | -0.3839 | 0.1029 | 0.2810 | -0.2810 | 0.9127 | 0.7638 | 0.2820 | -0.3647 |
| NO2-N-In-Rh@C60 | In61-O63 | 85 | 0.8513 | 0.4516 | -0.4977 | 0.4372 | 0.6056 | -0.6056 | 0.6622 | 0.3388 | 0.1452 | -0.1192 |
|  | In61-N63 | 73 | 0.7762 | 0.5833 | -0.5196 | 0.6052 | -0.8566 | 0.8566 | 0.2921 | 0.2206 | 0.3405 | -0.1178 |
|  | In61-C30 | 62 | 0.9276 | 0.3699 | -0.5007 | 0.3412 | 0.1591 | -0.1592 | 0.8768 | 0.4522 | 0.0568 | 0.6090 |
| NO2-O-In-Rh@C60 | In61-O63 | 63 | 0.6723 | 0.3832 | -0.3089 | 0.3433 | -0.3440 | 0.3440 | 0.5640 | 0.3076 | 0.0289 | -0.9273 |
|  | In61-O64 | 59 | 0.5434 | 0.2860 | -0.2381 | 0.2613 | -0.2315 | 0.2315 | 0.4619 | 0.3033 | 0.0095 | -0.6963 |
|  | In61-C54 | 29 | 0.2946 | -0.7509 | -0.4535 | 0.1329 | 0.3206 | -0.3206 | 0.8881 | 0.7380 | 0.2506 | -0.4638 |
| NO-N-In-Rh@C60 | In61-N62 | 75 | 0.4551 | 0.2168 | -0.1625 | 0.1693 | -0.6827 | 0.6827 | 0.7513 | 0.3288 | 0.0016 | -0.5629 |
|  | In61-C30 | 59 | 0.9723 | 0.3754 | -0.5340 | 0.3483 | 0.1857 | -0.1857 | 0.8964 | 0.4677 | 0.0601 | 0.6271 |
|  | In61-C53 | 74 | 0.9353 | 0.3605 | -0.4979 | 0.3296 | 0.1683 | -0.1683 | 0.8947 | 0.4665 | 0.0372 | -0.1234 |
| NO-O-In-Rh@C60 | In61-O63 | 93 | 0.3329 | 0.1796 | -0.1194 | 0.1478 | -0.2841 | 0.2841 | 0.4490 | 0.2444 | 0.0063 | -0.4131 |
|  | In61-C30 | 110 | 0.1022 | 0.3867 | -0.5795 | 0.3650 | 0.2145 | -0.2145 | 0.9042 | 0.4772 | 0.0647 | 0.6534 |
|  | In61-C53 | 81 | 0.9875 | 0.3788 | -0.5479 | 0.3534 | 0.1945 | -0.1945 | 0.8990 | 0.4706 | 0.0406 | -0.1323 |

**Table S2**: Spin State energies of In-Rh@C_60_ in different electronic configurations/states (eV)

| **System** | **QUINTET** | **SEPTET** | **TRIPLET** | **SINGLET** |
| --- | --- | --- | --- | --- |
| **In-Rh@C_60_** | 0.682 | 2.557 | 0 | 1.120 |

**Table S3.** Summary of the calculated descriptors for In-Rh@C_60_ and gas-adsorbed In-Rh@C_60_ calculated at DFT/PW6B95-D3/GenECP (Method 1) and ωB97X-D/LANL2DZ (Method 2) computational methods.

**Note:** All units are in electronvolst (eV), except for chemical softness (S) with a unit of eV^-1^

|  | Method 1 |  |  |  |  |  |  |  |  |  | Method 2 |  |  |  |  |
| --- | --- | --- | --- | --- | --- | --- | --- | --- | --- | --- | --- | --- | --- | --- | --- |
| Systems | IP | EA | µ | Η | S | ω | E_FL_ |  | IP | EA | µ | H | S | ω | E_FL_ |
| In-Rh@C_60_ | 5.641 | 4.187 | 4.914 | 0.727 | 0.688 | 16.606 | -4.914 |  | 7.662 | 3.681 | 5.672 | 1.991 | 0.251 | 8.080 | -5.672 |
| NH_3_-In-Rh@C_60_ | 5.088 | 3.569 | 4.329 | 0.760 | 0.658 | 12.334 | -4.329 |  | 6.419 | 1.629 | 4.024 | 2.395 | 0.209 | 3.380 | -4.024 |
| NO-N-In-Rh@C_60_ | 8.945 | 8.057 | 8.501 | 0.444 | 1.126 | 81.357 | -8.501 |  | 7.993 | 3.494 | 5.744 | 2.250 | 0.222 | 7.332 | -5.744 |
| NO-O-In-Rh@C_60_ | 8.676 | 7.972 | 8.324 | 0.352 | 1.419 | 98.354 | -8.324 |  | 7.115 | 3.963 | 5.539 | 1.576 | 0.317 | 9.734 | -5.539 |
| NO_2_-N-InRh@C_60_ | 9.876 | 8.722 | 9.299 | 0.577 | 0.866 | 74.907 | -9.299 |  | 8.539 | 3.374 | 5.957 | 2.582 | 0.194 | 6.870 | -5.957 |
| NO_2_-O-InRh@C_60_ | 9.591 | 8.054 | 8.823 | 0.769 | 0.650 | 50.627 | -8.823 |  | 8.458 | 3.435 | 5.947 | 2.512 | 0.199 | 7.040 | -5.947 |

**Table S4**: Thermodynamic parameters of the studied systems

| Parameters | In-Rh@C_60_ | NH_3_-In-Rh@C_60_ | NO_2_-O-In-Rh@C_60_ | NO-N-In-Rh@C_60_ | NO-O-In-Rh@C_60_ |
| --- | --- | --- | --- | --- | --- |
| ^Ɛ^0 | -64140.8 | -65705.2 | -69746.1 | -67699.4 | -67700.7 |
| ƐZPE | 10.3696 | 11.4305 | 10.5225 | 10.4484 | 10.4391 |
| Etot | 10.9354 | 12.0665 | 11.2334 | 11.1229 | 11.1704 |
| Hcorr | 10.9611 | 12.0922 | 11.2591 | 11.1486 | 11.1961 |
| Gcorr | 9.1363 | 10.1602 | 9.1287 | 9.0948 | 8.9852 |
| ^Ɛ^0 + ƐZPE | -64130.4 | -65693.7 | -69735.6 | -67689.0 | -67690.3 |
| ^Ɛ^0 + Etot | -64129.9 | -65693.1 | -69734.9 | -67688.3 | -67689.6 |
| ^Ɛ^0 + Hcorr | -64129.8 | -65693.1 | -69734.9 | -67688.3 | -67689.5 |
| ^Ɛ^0+ Gcorr | -64131.6 | -65695.0 | -69737.0 | -67690.3 | -67691.8 |
| ΔG^0^ | - | 15.1633 | 0.5455 | -0.0716 | -0.1208 |
| ΔH^0^ | - | 2.2176 | 0.0936 | -0.0523 | -0.0046 |


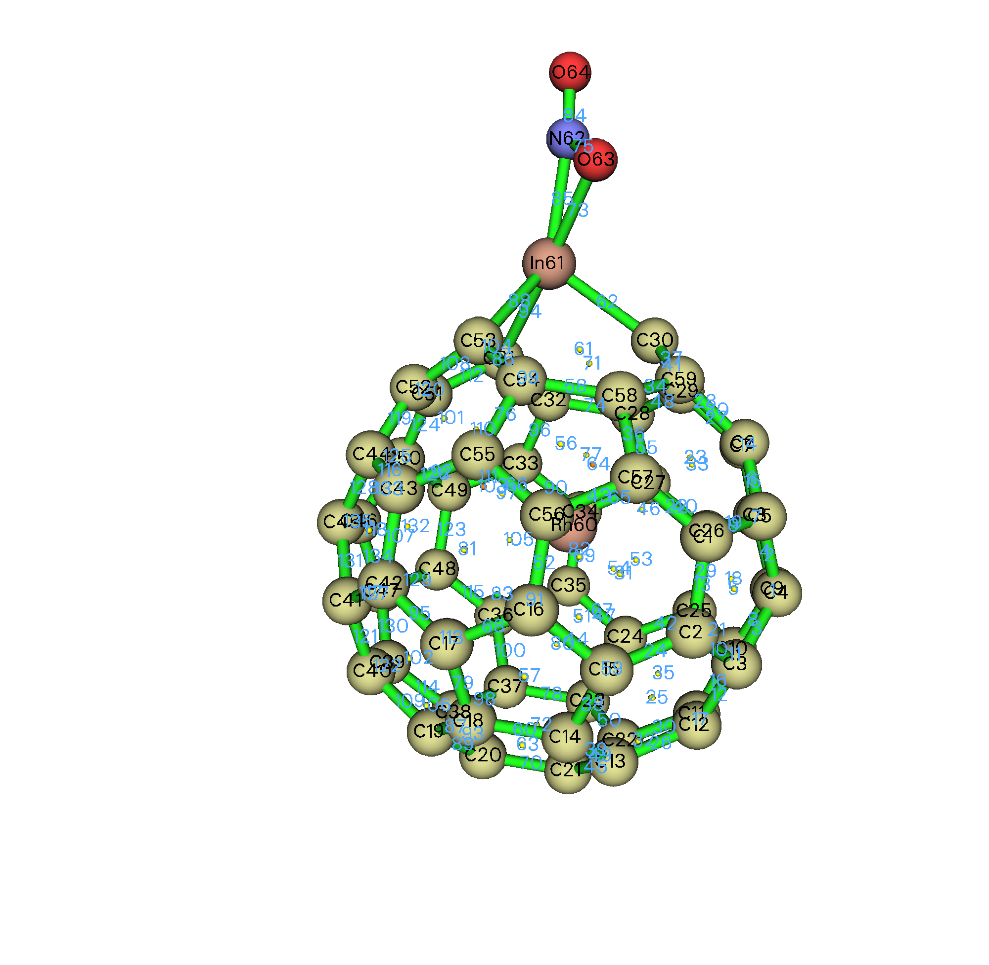

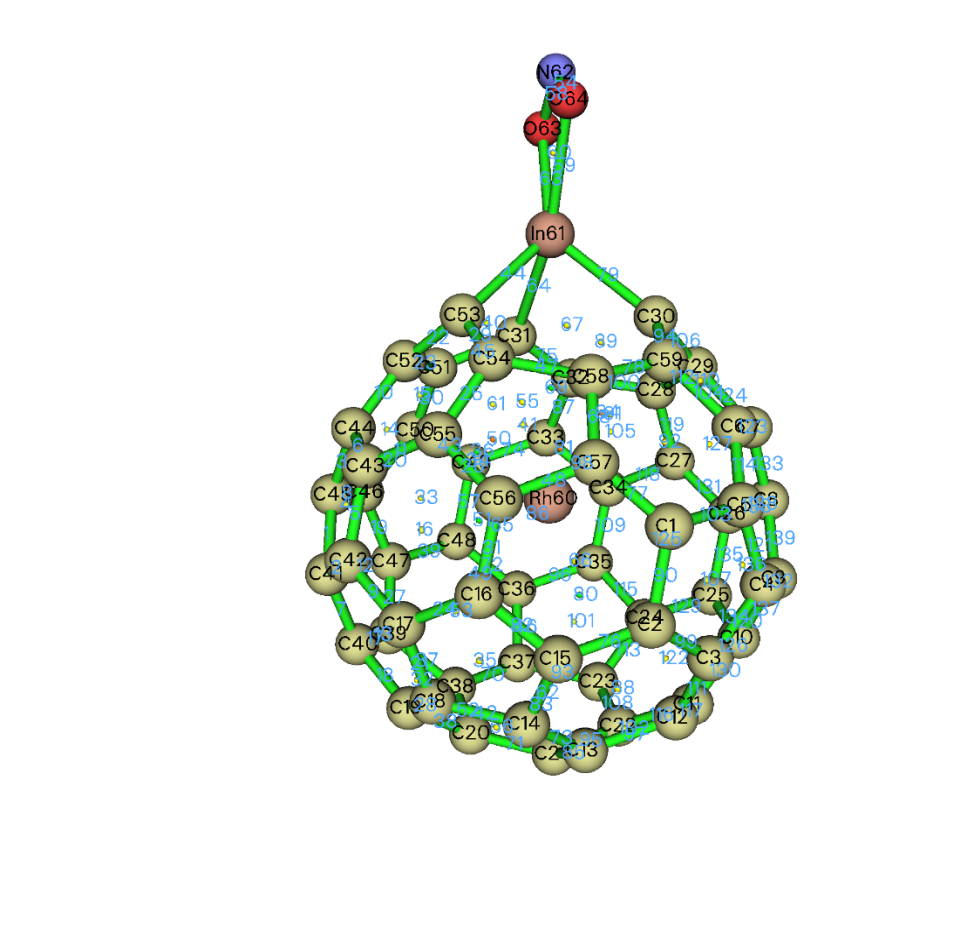

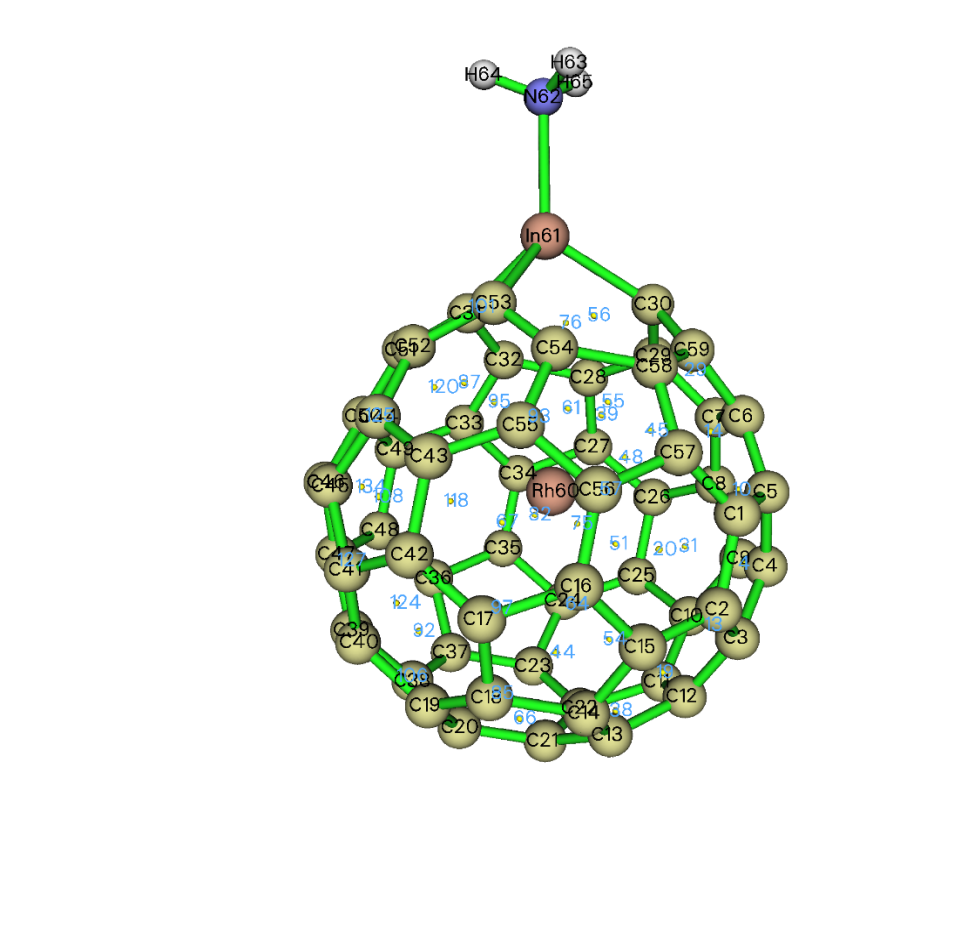


NO_2_-N-In-Rh@C_60_

NO_2_-O-In-Rh@C_60_

NH_3_-In-Rh@C60


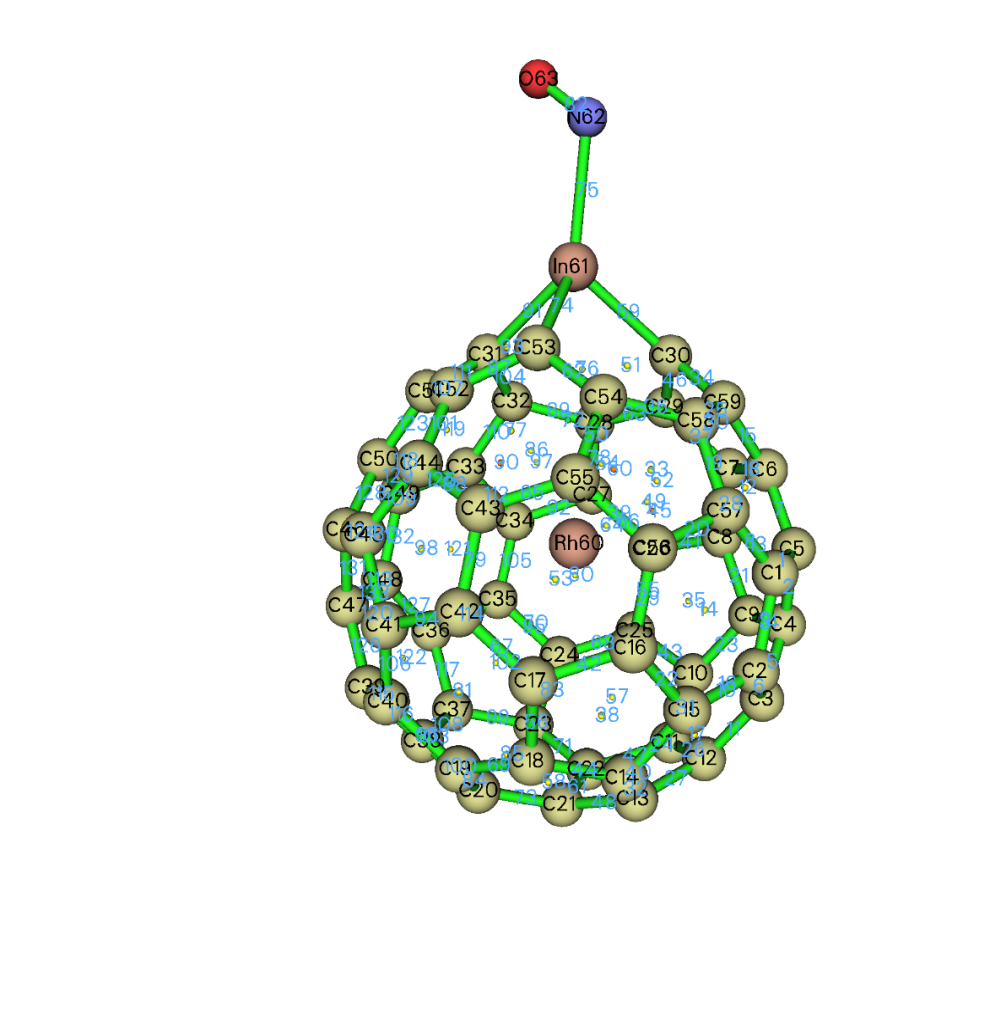

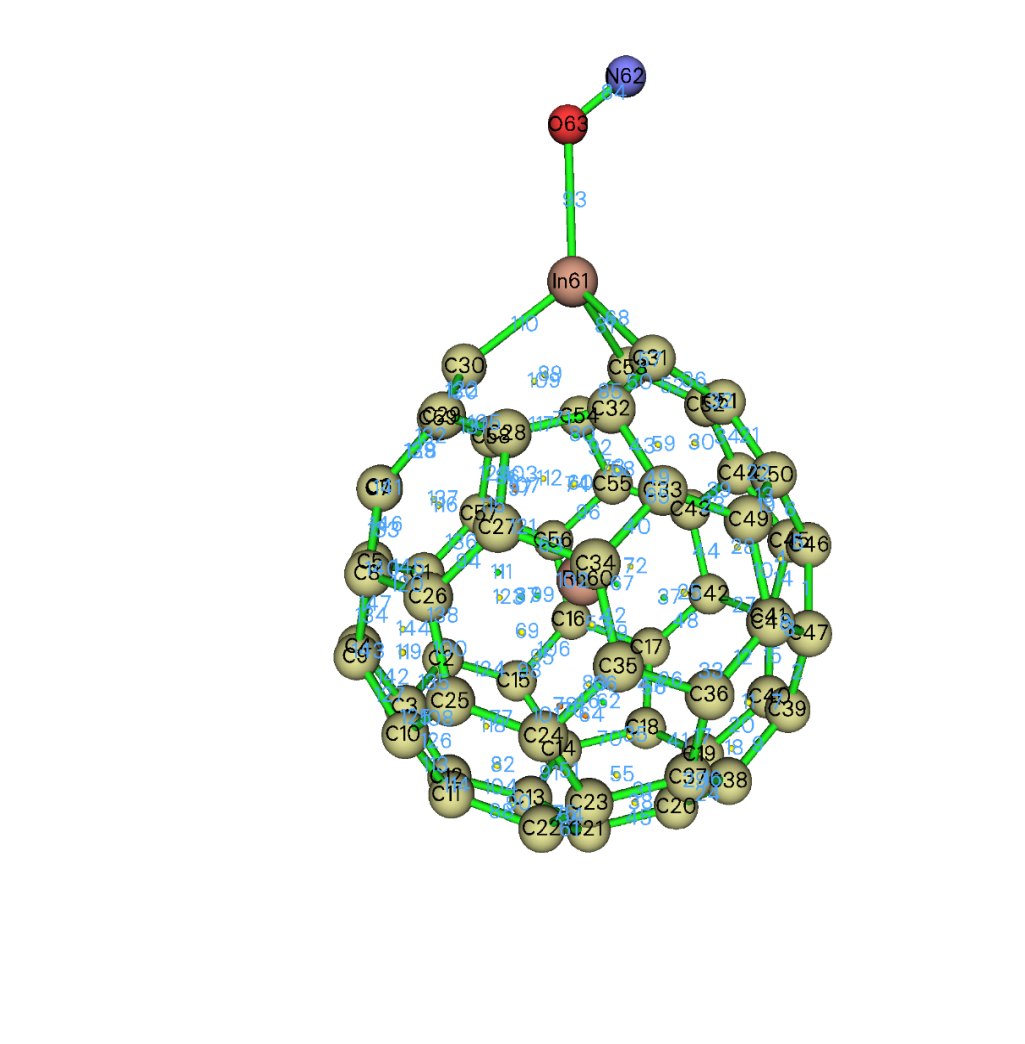


**CPs**

NO-O-In-Rh@C60

NO-N-In-Rh@C60

**Figure S1**: 3D plot for QTAIM analysis, indicating various critical points (CPs) for the studied systems
